# Improving the radical cure of vivax malaria (IMPROV): a study protocol for a multicentre randomised, placebo-controlled comparison of short and long course primaquine regimens 

**DOI:** 10.1186/s12879-015-1276-2

**Published:** 2015-12-07

**Authors:** The IMPROV Study Group

**Affiliations:** Global and Tropical Health Division, Menzies School of Health Research and Charles Darwin University, Darwin, 0810 NT Australia

**Keywords:** *Plasmodium vivax*, Primaquine, Short course, Long course, Radical cure

## Abstract

**Background:**

*Plasmodium vivax* malaria is a major cause of morbidity and recognised as an important contributor to mortality in some endemic areas. The current recommended treatment regimen for the radical cure of *P. vivax* includes a schizontocidal antimalarial, usually chloroquine, combined with a 14 day regimen of primaquine. The long treatment course frequently results in poor adherence and effectiveness. Shorter courses of higher daily doses of primaquine have the potential to improve adherence and thus effectiveness without compromising safety. The proposed multicentre randomised clinical trial aims to provide evidence across a variety of endemic settings on the safety and efficacy of high dose short course primaquine in glucose-6-phosphate-dehydrogenase (G6PD) normal patients.

**Design:**

This study is designed as a placebo controlled, double blinded, randomized trial in four countries: Indonesia, Vietnam, Afghanistan and Ethiopia. G6PD normal patients diagnosed with vivax malaria are randomized to receive either 7 or 14 days high dose primaquine or placebo. G6PD deficient (G6PDd) patients are allocated to weekly primaquine doses for 8 weeks. All treatment is directly observed and recurrent episodes are treated with the same treatment than allocated at the enrolment episode. Patients are followed daily until completion of treatment, weekly until 8 weeks and then monthly until 1 year after initiation of the treatment. The primary endpoint is the incidence rate (per person year) of symptomatic recurrent *P. vivax* parasitaemia over 12 months of follow-up, for all individuals, controlling for site, comparing the 7 versus 14-day primaquine treatment arms. Secondary endpoints are other efficacy measures such as incidence risk at different time points. Further endpoints are risks of haemolysis and severe adverse events.

**Discussion:**

This study has been approved by relevant institutional ethics committees in the UK and Australia, and all participating countries. Results will be disseminated to inform *P. vivax* malaria treatment policy through peer-reviewed publications and academic presentations. Findings will contribute to a better understanding of the risks and benefits of primaquine which is crucial in persuading policy makers as well as clinicians of the importance of radical cure of vivax malaria, contributing to decreased transmission and a reduce parasite reservoir.

**Trial registration:**

ClinicalTrials.gov Identifier: NCT01814683. Registered March 18, 2013

**Electronic supplementary material:**

The online version of this article (doi:10.1186/s12879-015-1276-2) contains supplementary material, which is available to authorized users.

## Background

*Plasmodium vivax* malaria is a major cause of morbidity and recognised as an important contributor to mortality in some endemic areas. Unlike *P. falciparum* malaria, *P. vivax* infections form dormant liver stages (hypnozoites) which cause relapses of the infection weeks to months after the initial attack. In South-East Asia relapse rates commonly exceed 50 %, making relapse the main cause of vivax illness. Recurrent episodes of febrile illness and haemolysis inflict a significant public health burden particularly in vulnerable groups such as pregnant women and young children. The first line treatment of vivax malaria is a combination of chloroquine (providing blood schizontocidal activity), and primaquine (providing liver hypnozoitocidal activity). Primaquine, an 8 aminoquinoline, is currently the only licensed drug with activity against hypnozoites. An important constraint on the global deployment of primaquine is its potential to cause haemolysis in patients with glucose-6-phosphate dehydrogenase deficiency (G6PDd), which typically occurs in 2–15 % of patients in endemic regions [1]. Individuals with less than 10 % of G6PD enzyme activity are at risk of life-threatening haemolysis [2] whereas the haemolysis in those with milder variants may be negligible [3]. In practice the lack of available robust diagnostics for G6PDd, concerns over drug toxicity, and the misperceived benign nature of *P. vivax* infection results in healthcare providers rarely prescribing primaquine even when recommended in policy. The lack of a safe and reliable radical cure of *P. vivax* is a major threat to current malaria control and elimination efforts.

The main determinant of primaquine efficacy is the total dose of primaquine administered, rather than the dosing schedule [3]. The 14 day regimen was chosen to reduce the required daily dose to mitigate the risk of haemolysis, which is related to the individual dose administered. Previous trials have demonstrated that the standard low dose regimen of primaquine (3.5 mg/kg total, amounting to 15 mg once daily in adults) fails to prevent relapses in many different endemic locations [4]. For this reason the 2010 WHO antimalarial guidelines now recommend a high dose regimen of 7 mg/kg (equivalent to an adult dose of 30 mg per day), although many countries still recommend lower doses for fear of causing more serious harm to unscreened G6PDd patients.

Shorter courses of higher daily doses of primaquine have the potential to improve adherence and thus effectiveness without compromising efficacy [3]. Primaquine also has relatively weak but clinically relevant asexual stage activity against *P. vivax* so larger daily doses may substantially augment chloroquine’s blood stage activity in areas of low level chloroquine resistance. In Thailand directly observed primaquine (1 mg/kg/day) administered over 7 days was well tolerated and reduced relapses by day 28 to 4 % [5]. This is encouraging but not definitive since many relapses present after one month, hence studies with longer follow-up are needed to distinguish whether relapse was prevented or deferred. If the efficacy, tolerability and safety of short-course, high-dose primaquine regimens can be assured across the range of endemic settings, along with reliable point-of-care G6PDd diagnostics, then this new primaquine regimen would be a major advance in malaria treatment improving adherence to and thus the effectiveness of anti-relapse therapy.

The radical cure of *P. vivax* in patients with known G6PDd is challenging. Current WHO guidelines recommend a weekly dose of 0.75 mg/kg for 8 weeks which mitigates primaquine-induced haemolysis [6] whilst retaining efficacy [7]. The weekly dosing schedule was derived from studies in the USA in a small number of healthy adults with the mildly primaquine-sensitive African A- G6PDd variant. Since host vulnerability to haemolysis varies between the over 100 different G6PDd variants [8], the available evidence is inadequate to ensure the universal safety of a 0.75 mg/kg dose either as a single dose, as advocated for reducing the transmission of falciparum malaria, or a weekly dose for the radical cure of vivax malaria [9].

Due to the long duration of standard primaquine treatment regimens, courses are difficult to supervise, are poorly adhered to and lack effectiveness. The proposed multicentre randomised clinical trial will provide evidence across a variety of endemic settings on the safety and efficacy of high dose-short course primaquine in G6PD normal patients. In a parallel single arm study data on the safety of weekly primaquine in patients with G6PDd will be obtained. The study aims to generate evidence that will directly inform global public health policy for the radical cure of *P. vivax*. A better understanding of the risks and benefits of primaquine is crucial in persuading policy makers and clinicians of the importance of the radical cure of vivax malaria that will reduce the parasite reservoir and decrease transmission. The strength and limitations of this study are listed in Table 1.

**Table 1:** Strengths and limitations of this study

• The IMPROV Study is a multi-centre study in different regions providing evidence of primaquine tolerability across a variety of endemic settings.
• The long follow up (12 months) and continuation of follow up through multiple recurrences, allows estimation of the incidence density of all episodes, which is a better indicator of the overall morbidity of *P. vivax* relapse.
• A major challenge in estimating the efficacy of primaquine comes from our inability to distinguish relapses from new *P. vivax* infections. The control arm receiving the placebo is critical in providing provide comparative data from which to estimate the efficacy of the primaquine regimens.
• The trial assumes that a shorter course of primaquine will increase adherence and therefore effectiveness, however this not be tested directly in this clinical study

The primary objective of this study is therefore to determine whether a 7-day primaquine regimen is safe and not inferior to the standard 14-day regimen (total dose of 7 mg/kg in both arms) in preventing *P. vivax* relapse in G6PD normal patients. Secondary objectives are to assess the absolute risks and benefits of radical treatment regimens in different endemic settings, to provide data on the safety of a weekly dose of primaquine (0.75 mg base/kg) in patients with G6PD deficiency and to identify the most cost-effective strategies for the management of *P. vivax* with respect to the use of G6PD tests, the dosing schedule and the epidemiological context.

## Study design & methods

### Summary of trial design

This is a randomized, double-blind, placebo-controlled, non-inferiority trial in G6PD normal patients with uncomplicated vivax malaria in seven participating study sites in Indonesia (two sites), Vietnam, Ethiopia (2 sites) and Afghanistan (two sites). Patients presenting to a participating treatment centre with uncomplicated vivax malaria and fulfilling the enrolment criteria will be randomly assigned to one of three treatment arms:

- ○ Intervention 1: Standard blood schizontocidal therapy plus 14 days of supervised primaquine (7 mg/kg total dose) administered once per day (0.5 mg/kg).
- ○ Intervention 2: Standard blood schizontocidal therapy plus 7 days of supervised primaquine (7 mg/kg total dose) administered once per day (1.0 mg/kg OD) followed by 7 days of placebo.
- ○ Control arm: Standard blood schizontocidal therapy plus 14 days placebo.

Patients tested initially and found to be G6PD deficient will be excluded from the randomised study but offered enrolment in a single arm non-randomised observational study to receive standard schizontocidal therapy plus primaquine 0.75 mg/kg/week for 8 doses (total dose 6 mg/kg).

All patients will receive standard medical care for the management of uncomplicated malaria, with blood schizontocidal treatment administered as either chloroquine (total dose 25 mg base/kg) or an artemisinin combination therapy depending on local recommendations and known chloroquine efficacy.

Recurrences of any species within 28 days will be considered treatment failures and treated with local second line alternatives (such as ACT or 7 days quinine) according to national guidelines. After 28 days, treatment failure is less likely and so patients will be treated with the same treatment regimen as that allocated at enrolment. Primaquine/placebo will be administered with food (crackers or a biscuit), which has been shown to reduce gastrointestinal side effects. All doses of study drugs will be supervised. If participants cannot visit the study centre, or fail to attend during the 14 days of supervised therapy, team members will visit them in their homes or places of school or work to ensure complete dosing.

Treatment efficacy and patient safety will be ensured by close monitoring over a 12 months follow up period following a schedule of visits and corresponding clinical and laboratory examinations. Each recurrent *P. vivax* will be confirmed by microscopy and recorded. Intercurrent *P. falciparum* parasitaemia will be treated with the locally recommended ACT .

### Trial participants

Male and female patients over 6 months of age presenting to a participating treatment centre with uncomplicated vivax malaria will be enrolled into the study if they fulfil the following inclusion and exclusion criteria. All patients will be screened for G6PD status using the NADPH spot test and those found to be deficient will be excluded from the main trial but encouraged to enrol in a parallel G6PDd arm. Participants in this non-randomised, observational study will receive the standard blood schizontocidal therapy plus a single supervised weekly dose of primaquine 0.75 mg base/kg weekly for 8 weeks (6 mg/kg total dose), with a similar follow up regimen as those patients in the primary study.

### Inclusion criteria

The participant may enter the study if ALL of the following apply:

- ○ Participant (or parent/guardian of children below age of consent) is willing and able to give written informed consent to participate in the trial; verbal consent in the presence of a literate witness is required for illiterate patients. In addition, written assent (or verbal assent in the presence of a literate witness for illiterates) from children 12 to 17 years as per local practice.
- ○ Monoinfection with *P. vivax* of any parasitaemia in countries which use CQ as blood schizontocidal therapy. Mixed infections with *P. vivax* and *P. falciparum* can be enrolled in countries which use an artemisinin combination therapy.
- ○ Diagnosis of malaria can be based on either malaria rapid diagnostic test or slide microscopy, as per site preference.
- ○ Over 6 months of age
- ○ Weight 5 kg or greater
- ○ Fever (axillary temperature ≥37.5 °C) or history of fever in the last 48 h.
- ○ Able, in the investigator’s opinion, and willing to comply with the study requirements and follow-up.

### Exclusion criteria

The participant may not enter the study if ANY of the following apply:

- ○ Female participant who is pregnant or lactating
- ○ Inability to tolerate oral treatment
- ○ Previous episode of haemolysis or severe haemoglobinuria following primaquine
- ○ Signs/symptoms indicative of severe/complicated malaria or warning signs requiring parenteral treatment
- ○ Haemoglobin concentration less than 9 g/dL
- ○ Known hypersensitivity or allergy to the study drugs
- ○ Blood transfusion in last 90 days, since this can mask G6PD deficient status
- ○ Presence of any condition which in the judgment of the investigator would place the participant at undue risk or interfere with the results of the study (e.g. serious underlying cardiac, renal or hepatic disease; severe malnutrition; HIV/AIDS; or severe febrile condition other than malaria)
- ○ Coadministration of other medication known to cause haemolysis
- ○ Currently taking medication known to interfere significantly with the pharmacokinetics of primaquine and the schizontocidal study drugs
- ○ Previously been a study participant in IMPROV (i.e. the same patient cannot be enrolled twice)

### Study flow

#### Screening

When malaria is suspected, a thick and thin blood smear will be obtained for microscopic diagnosis of *P. vivax*. Some sites will also routinely use rapid diagnostic tests to complement microscopy. After the diagnosis is confirmed microscopically or by Rapid Diagnostic Test (RDT), the patient will be approached for informed consent. In some centres, the following tests are considered part of clinical practice: malaria blood film, RDT, G6PD test and haematocrit or haemoglobin concentration. If any one of these tests is not routine, then consent and assent will be obtained before these tests are performed.

#### Informed consent

The participant (or parent/guardian of children below age of consent) must personally sign and date the latest approved version of the informed consent form before any study specific procedures are performed. Alternatively, verbal consent in the presence of a literate witness will be obtained from illiterate patients. In addition to the Informed Consent, Assent will be obtained from children 12 to 17 years of age if locally required.

#### Study procedures (Table 2)

**Table 2:** Study schedule

	Day																Week						Month										Rec	Age > 60 m			Age 12–60 m			Age 6–< 12 m		
	0	1	2	3	4	5	6	7	8	9	10	11	12	13	14^a^	17^a^	3	4	5	6	7	8	3	4	5	6	7	8	9	10	11	12		vol	freq	total	vol	freq	total	vol	freq	total
History of fever, current axillary temperature	X	X	X	X	X	X	X	X	X	X	X	X	X	X	X	X	X	X	X	X	X	X	X	X	X	X	X	X	X	X	X	X	X									
Full medical history, vital sign, physical examination	X																																									
MUAC & weight	X																						X			X			X			X	X									
Ask about any concomitant medication	X	X	X	X	X	X	X	X	X	X	X	X	X	X	X	X	X	X	X	X	X	X	X	X	X	X	X	X	X	X	X	X	X									
Symptom checklist	X	X	X	X	X	X	X	X	X	X	X	X	X	X	X	X	X	X	X	X	X	X	X	X	X	X	X	X	X	X	X	X	X									
Asses for any indication for study withdrawal		X	X	X	X	X	X	X	X	X	X	X	X	X	X	X	X	X	X	X	X	X	X	X	X	X	X	X	X	X	X	X	X									
Interviews to asses cost-of-illness	X													X	X																		X									
Urine pregnancy test	X																																X									
Masimo puls-oximeter-main study	X	X	X	X				X						X																												
Masimo puls-oximeter-G6PDd arm	X	X	X	X				X							X																											
Drug treatment																																										
Standard schizontocidal theraphy	X	X	X																														X									
PQ-main study	X	X	X	X	X	X	X	X	X	X	X	X	X	X																			X									
PQ-G6PDd arm	X							X							X		X	X	X	X	X												X									
Capillary sample-microtainer up to 400 μL																																		0.4	26	10.4	0.4	26	10.4	0.4	26	10.4
G6PD flourescent spottest^b^	X																																									
G6PD rapid test	X																																									
Parasite microscopy^b^	X	X	X	(X)	(X)	(X)	(X)	(X)									X	X	X	X	X	X	X	X	X	X	X	X	X	X	X	X	X									
Rapid diagnostic test for malaria^b^	X																																									
Haemoglobin HemoCue^b^-main study	X			X				X						X			X	X	X	X	X	X	X	X	X	X	X	X	X	X	X	X	X									
Haemoglobin HemoCue^b^-G6PDd arm	X			X				X			X				X	X	X	X	X	X	X	X	X	X	X	X	X	X	X	X	X	X	X									
Serology	X																	X				X	X			X			X			X	X									
Parasite genotype	X																																X									
EDTA venous sample^c^																																										
From D0 EDTA: FBC, G6PD, HP, PG, serol, DC	X																																	5	1	5	2	1	2	0.5	1	0.5
From D7 & 13: FBC, DC								X					X	X																				5	2	10	2	2	4	0.5	2	1
EDTA Month 6: FBC, G6PD, pPCR, Serol																										X								5	1	5	2	1	2	0.5	1	0.5
EDTA each recurrence: FBC PG, Serol, DC																																	X	5	2	10	2	2	4	0.5	2	1
Total blood volumes-2 malaria recurrence																																				40.4			22.4			13.4
Total blood volumes-no recurrence malaria																																				30.4			18.4			12.4

^a^G6PDd arm only

^b^in some centres these tests are routine & do not need to be repeated

^c^if a venous sample cannot be obtained, obtain a capillary sample into a Microtainer

**Demographics:** The patient’s date of birth (or if not known, the estimated age) and gender will be recorded. If the patient is a female of childbearing age, she will be asked if she is currently pregnant, lactating, planning to get pregnant and the date of the first day of her last menstrual period. These questions will be culturally adapted in countries with strict prohibitions and restrictions on marriage and pregnancy.

**Pre-treatment temperature:** The patient (or parent/guardian of children) will be asked if he/she had fever during the past 48 h and his/her current temperature (axillary) will be measured. This will be done on screening and each subsequent visit.

**Medical history and physical examination:** The details of any disease/surgical conditions, and drug allergies will be recorded. The patient’s pulse rate, respiratory rate, weight, mid upper arm circumference (MUAC) in children < 5 years, and the results of a baseline physical examination will be recorded. This will be done on screening and each subsequent visit.

**Prior medication in the last 28 days:** All over-the-counter or prescription medication, vitamins, and/or herbal supplements will be recorded. This will include the medicine name (generic name, if known), starting and ending dates, total dose, route of administration and the indication for use. This will be done on screening and each subsequent visit.

**Capillary blood sample (up to 400 μl)** will be obtained at enrolment for the following tests (if not already done as part of routine care):

- ○ *Field Blood Haemoglobin*. Samples will be obtained at the initial visit, Day 3 and each weekly or monthly follow up visit (Hemocue™ system, Angelholm, Sweden).
- ○ *Rapid Diagnostic test (RDT) for malaria*. A multi-species (pf-HRP2/pan-pLDH and pf-HRP2/aldolase) RDT for the diagnosis of falciparum and vivax malaria will be undertaken on initial screening. The decision whether to enrol a patients is based on the RDT result.
- ○ *Parasite microscopy.* A thick and thin malaria smear will be made at each scheduled visit (or at least until 2 negative malaria smears) and at any unscheduled visit if malaria is suspected. Microscopy will be used to confirm the presence of parasitaemia and to estimate the parasite density.
- ○ *G6PD Testing.* Blood will be collected in an EDTA tube or heparinised haematocrit capillary tube on initial screening for glucose-6-phosphate-dehydrogenase semi-quantitative fluorescent spot test for initial screening in the field. A reagent solution containing Glucose-6-P + NADP+ is mixed with whole blood or a dried blood spot. Samples obtained from normal or slightly reduced G6PD activity will show strong fluorescence. Failure to fluoresce after 10-min of incubation suggests a total or marked deficiency of G6PD. This test may fluoresce falsely if the study participant has had a blood transfusion within the last 90 days. Definition of G6PD status for the purpose of enrolment will be decided based on the NAPD Spot test. If the result is deficient or borderline, the patient will be enrolled into the G6PD deficient arm
- ○ *Other tests* on remaining capillary blood include the following:
  - ▪ Parasite genetic studies (e.g. molecular marker for drug resistance, parasite diversity and distinguishing recurrent parasitaemias). These will be used to explore parasite factors associated with recurrent *P. vivax* infection.
  - ▪ Host genotyping for G6PD and other red cell and cytochrome P450 drug metabolism polymorphisms (such as 2D6). These will be used to explore whether host factors influence primaquine blood concentrations which may affect treatment failure.
  - ▪ Drug concentrations (CQ, PP, PQ & carboxy PQ).
  - ▪ Quantitative analysis of G6PD status will be carried out at a suitable reference centre.
  - ▪ Repeat G6PD testing with novel G6PD tests. To assess the potential of rapid point of care diagnostic tests.
  - ▪ Serology, to assess the immune status of the patient and whether this affects symptomatic disease.

**A venous blood sample** will be obtained on enrolment, days 7 and 13/14 (but only the first episode of malaria), the first day of each subsequent recurrence, and at a mid-term review at 6 months for the following tests:

- ○ *Complete Blood Count (CBC)*. Automated CBCs will be analysed using the Sysmex pocH-100i™, or equivalent, machine, if the machine is available.
- ○ *Pharmacokinetic Analysis (PK).* Blood samples will be collected on day 7 and day 13/14 for HPLC analysis of blood concentrations including CQ, PP, primaquine and carboxyprimaquine.
- ○ *Other tests* on remaining venous blood include the following:
  - ▪ Parasite genetic studies (e.g. molecular marker for drug resistance, parasite diversity and distinguishing recurrent parasitaemias). These will be used to explore parasite factors associated with recurrent *P. vivax* infection.
  - ▪ Host genotyping for G6PD and other red cell and cytochrome P450 drug metabolism polymorphisms (such as 2D6). These will be used to explore whether host factors influence primaquine blood concentrations which may affect treatment failure.
  - ▪ Flow cytometry – to detect the G6PD activity in heterozygous women.
  - ▪ Drug concentrations (CQ, PP, PQ & carboxy PQ)
  - ▪ G6PD quantitative assay in sites where laboratory facilities permit timely sample processing (if not done on capillary blood). This will be used to explore whether the degree of G6PD activity influences the side effect profile.
  - ▪ Serology, to assess the immune status of the patient and whether this affects symptomatic disease.

The total amount of blood to be collected during the study period of 12 months will vary depending on the number of recurrent episodes of malaria. If there are no recurrences, the minimum blood volumes will be approximately 31 mL (patient aged > 5y), 19 mL (age 12–60 m) and 13 ml (age 6–< 12 m). If there are two recurrences, these volumes become approximately 41, 23 and 14 mL. These volumes anticipated over a 12 month follow up are well within the acceptable limits for patients aged 6 months to 14 years.

Blood for protocol mandated tests that cannot be done straight away, will be stored for future analysis. This may result in unused blood remaining. Permission will be sought for the long term storage of this unused blood for future tests relevant to the study outcomes. The length of storage will follow local ethics committee guidelines. Any new tests will need ethical approval. Any transfer of specimens will follow all relevant national and IATA regulations.

**Urine β-HCG pregnancy test** will be performed in all women of childbearing age (unless menstruating) eligible for enrolment. The decision to perform the test in unmarried women will be culturally adapted and follow local practice in countries with strict prohibitions and restrictions on marriage and pregnancy. Bioline HCG test strips or an equivalent test will be used. After the initial screening, the test will be performed during scheduled and unscheduled visits if recurrent *P. vivax* or *P. falciparum* is diagnosed.

#### Regiment allocation

In participating centres, where the G6PD result is available straight away, regimen allocation and administration of the study agent will be on Day 0. However, in participating centres where the G6PD result is not available on the day of enrolment, regimen allocation and administration of the study drugs will be on Day 1. Participating centres can decide whether they prefer to give PQ on Day 0 immediately following schizontocidal therapy or on Day 1 following the start of the schizontocidal therapy. It is important that once a decision is made for each site there will be consistent adherence to the chosen starting day within that site.

Once G6PD results are available, those testing normal will be randomized in the 3-arm main study whereas, G6PD deficient participants will be enrolled into an observational (non-randomised) open-label arm.

#### Randomization

A randomization list for participants will be prepared centrally at the Mahidol-Oxford Tropical Medicine Research Unit (MORU), Bangkok, Thailand. Randomisation will be in blocks of 20 for each dosing band. Individual patients drug kits containing primaquine/placebo drug cards will be labelled by weight bands: A – 5–34 kg, B - 35–45 kg, and C- ≥ 46 kg. There will be 20 individual patient drug kits in a box.

The study staff responsible for generating the randomization list and for selecting code letters for the study agents will not be involved in any other way in the conduct of the trial and will not be present at the study site.

Randomisation to determine the regimen allocation will be carried out as soon as a participant is enrolled and will be based on his/her weight. The first drug kit in the relevant box will be given to the first patient; the second drug kit will be given to the next patient in that weight category and so on. The number on the drug kit will be the unique drug randomisation number. It will be recorded onto the CRF as well as the subject number.

#### Blinding

Primaquine and primaquine placebos have been manufactured specifically for the study. In order to conceal the allocation of primaquine versus placebo and dose regimen of primaquine, the study will use 7.5 mg and 15 mg tablets primaquine and a placebo with similar appearance. Primaquine will be administered as a single daily dose: 0.5 mg/kg/day × 14 days or 1.0 mg/kg/day × 7 days.

#### Medication for blood stage infection

All study participants will receive blood schizontocidal treatment administered as either chloroquine (total dose 25 mg base/kg) or an artemisinin based combination treatment (ACT), depending on local recommendations and known chloroquine efficacy. Chloroquine (each tablet containing 155 mg of base) will be given at initially (4 tablets or 10 mg/kg for children) and then at 6–8, 24 and 48 h (2 tablets or 5 mg/kg for children). Artekin™ (each tablet containing 40 mg dihydroartemisinin and 320 mg piperaquine) will be given at 0, 24 and 48 h. Artemether-lumefantrine (CoArtem®) is another ACT in use in some countries. One tablet contains 20 mg of artemether and 120 mg of lumefantrine. Dosing will be by weight administered twice daily for three days.

#### Study treatment

Primaquine is an 8-aminoquinoline with cidal activity against the gametocytes and hypnozoites of *P. vivax*. It is essentially a pro-drug that is extensively and rapidly metabolized to carboxyprimaquine with only a small fraction of the parent drug excreted unchanged. However, little is known of the pharmacokinetics of the metabolites that are responsible for both its antimalarial activity and toxicity. Its principal metabolite, carboxyprimaquine, is formed as a result of oxidative deamination, which is thought to involve both the cytochrome-P450 enzyme complex and monoamine oxidases (MAO). Primaquine is rapidly and almost completely absorbed following oral administration, with peak plasma concentrations (Cmax) reached within 3 h. It is cleared by hepatic biotransformation, with an elimination half-life of 8 h. The pharmacokinetics of primaquine does not seem to be time-dependent, showing similar kinetics after repeated dosages. The most important adverse effect is acute haemolytic anaemia in patients with G6PD deficiency. Primaquine may also cause abdominal pain if administered on an empty stomach. Other side effects include nausea and vomiting. Uncommon effects include mild anaemia and leucocytosis. Primaquine causes methaemoglobinaemia (levels of up to 18 % are reported, normal level is <1 %), but this seldom causes symptoms in healthy individuals and is self-limiting.

Eligible G6PD-normal patients will be randomized to two primaquine regimens and the control arms in a ratio of 2:2:1.

- ○ Primaquine regimen 1: 14 days of supervised primaquine (7 mg/kg total dose) administered once per day (0.5 mg/kg)
- ○ Primaquine regimen 2: 7 days of supervised primaquine (7 mg/kg total dose) administered once per day (1.0 mg/kg OD) followed by 7 days of supervised placebo
- ○ Control Arm: 14 days of supervised placebo

#### G6PD deficient arm

Eligible G6PD deficient participants will be enrolled into an observational (non-randomised) open-label arm. Aside from standard blood schizontocidal therapy, these participants will receive a single supervised weekly dose of primaquine 0.75 mg base/kg weekly for 7 weeks i.e. 8 doses (6 mg/kg total dose). During an 8-week treatment regimen haematological screening and clinical assessments will be made prior to each weekly dose of primaquine. Haemoglobin concentration will be assessed using HemoCue™ (Angelholm, Sweden) done on Days 3, 7, 10, 14, 17, 21 etc. in the G6PDd arm. The proportion of subjects completing all 8 doses will be classified as “safely treated”. Subjects deemed at risk with further dosing will not be treated further with primaquine. Providing that consent is not withdrawn, all patients, irrespective of completion of therapy, will continue to be followed up to document safety and efficacy parameters at 12 months.

#### Vomiting

All patients in either group will be observed for one hour after treatment. If vomiting occurs within one hour, the investigator has the option of redosing with both drugs or omitting the primaquine/placebo or open label primaquine. If the repeat dose of ACT / CQ is also vomited within one hour, the patient will be stopped oral treatment and will be given parenteral rescue treatment.

When the patient is able to eat and drink normally, they will continue with their study drugs to complete their courses of both the schizonticidal treatment and the primaquine/placebo or open primaquine. Drugs which have been vomited will be counted as administered treatment on that day.

Patients may develop persistent vomiting, even after the acute phase of illness. In this case the primaquine/placebo will be stopped and restarted once the vomiting has resolved.

#### Subsequent assessments

Patients will be seen daily until PQ treatment is completed, weekly until week 8 and then monthly until 1 year after enrolment. For the weekly visits a window of 3 days either side will be acceptable for the scheduled follow up. For the monthly visits, 7 days either way will be acceptable. Some subjects may be unable to attend the clinic or be away from home. If such patients own a mobile phone, they can be contacted and interviewed by phone to complete a symptom questionnaire to assess safety outcomes. The most important adverse effect of primaquine is haemolytic anaemia in patients with G6PD deficiency. Haemoglobin concentration (from finger prick sampling by HemoCueAB™, Angelholm, Sweden) will therefore be checked regularly.

Methaemoglobinemia will be assessed using a non-invasive oximeter (Masimo) at baseline, daily for the first three days then D7 and 13/14. MetHb will be measured in a subgroup of patients at two selected sites in the first instance; thereafter, the Masimo machine will be moved to another site. To reduce the risk of unblinding of patient allocation, metHb concentrations will be measured by a research team member who will not be involved in other patient assessments. These data will be recorded in a register and not on the case report form to reduce the risk of unblinding the treatment regimen.

#### Recurrent episodes of malaria

Participants will attend the study centre according to the scheduled times and will also be encouraged to report to the study centre in the event of any illness. The blood film will only be read immediately if the patient is symptomatic (i.e. has a fever or a history of fever in the preceding 24 h); this mirrors the real world scenario of passive case detection when patients only present to a clinic if they are sick.

If the patient is well at a routine follow up the blood film will be stored and processed at a later date in the reference laboratory. This will provide data on asymptomatic parasitaemia and how often it resolves spontaneously; this has important implications for understanding the transmission of malaria back to the community. Asymptomatic patients subsequently found to have peripheral parasitaemia will not be sought and treated.

#### Rescue treatment

Treatment will only be offered to patients who are symptomatic patients.

- ○ A recurrent vivax parasitaemia within 28 days is potentially a recrudescent infection. These patients will be treated according to national guidelines, usually an ACT or quinine. They will remain in the study, have their parasitaemia checked on Day 7 and will then resume routine follow up.
- ○ After 28 days, a recurrent vivax parasitaemia is more likely to represent a relapse. These patients will be treated with a repeat supervised course of the same regimen that was administered on Day 0 (i.e. the same schizonticidal drug and primaquine/placebo) with additional follow-up on days 7 and 14, and thereafter at their scheduled monthly follow up visit until the end of the study.
- ○ Those in the G6PDd group will also be treated with the same schizonticidal drug administered on Day 0 and will either complete their 8 doses of primaquine, if this has not been completed when the recurrence occurred or will be given a new course of 8 doses, if not on primaquine at the time of the recurrence.
- ○ If patients develop symptomatic malaria with *P. falciparum* parasitaemia (either alone or mixed with *P. vivax*), they will be treated according to national guidelines (usually an ACT). Some countries also recommend a single dose of primaquine 0.45 mg/kg for transmission blocking. Patients who develop severe malaria will be admitted to hospital and treated with the recommended parenteral drug. Some CQ treated patients may develop falciparum parasitaemia while still taking CQ, if this happens, they will be treated with an ACT.

Patients with recurrent malaria either due to *P. vivax* or *P.falciparum*, will have their treatment supervised and clinical and parasitological recovery documented and continue in the study.

#### Maximum number of symptomatic vivax recurrences

Patients may experience symptomatic recurrent vivax parasitaemia during follow up after they have received treatment for their initial *P. vivax* infection. Patients may experience multiple episodes of *P. vivax* infection. Each episode will be documented and the patient retreated with the study drug. If a patient has more than four symptomatic recurrences of *P. vivax*, outside the time defined for a recrudescent infection, then they will not be prescribed any further study drug. Patients on their fifth or subsequent symptomatic *P. vivax* recurrence will be treated with supervised open primaquine at a dose of 0.5 mg/kg × 14 days. They will remain in the study and be followed up to Day 365.

In Vietnam, retreatment with study drug will only be allowed for three symptomatic *P. vivax* recurrences within the first six months of the study. Such patients will then be treated with be treated with supervised open primaquine at a dose of 0.5 mg/kg × 14 days. They will remain in the study and be followed up to Day 365.

At the end of the study, patients who have had 1 or more *P. vivax* recurrences will be treated with supervised open primaquine at a dose of 0.5 mg/kg × 14 days, if this has not already been given. Those patients who did not have any recurrent vivax parasitaemias are at low risk of further relapse, and will not be offered supervised open primaquine.

#### Concomitant treatments

Throughout the study investigators may prescribe other concomitant medications or treatments deemed necessary to provide adequate supportive care, e.g. paracetamol for fever. Any medication, other than the study medication taken during the study will be recorded in the CRF. Drugs conferring a risk of hemolysis are listed in table 3.

**Table 3:** Drugs conferring risk of haemolysis

Potentially haemolytic drugs	Drugs with possible risk of haemolysis	drugs with doubtful risk of haemolysis	Drugs that may increase the exposure of primaquine, chloroquine, piperaquine and lumefantrine metabolism by inhibiting the cytochrome (CYP) 450:	The following drugs may decrease the exposure of primaquine, chloroquine, piperaquine and lumefantrine metabolism by inducing CYP 450:
Dapsone	Ciprofloxacin	Chloramphenicol	Ketoconazole	Barbiturates
Sulphonamides	Norfloxacin	Quinine	Itraconazole	Carbamazepine
Methylene blue	Chloroquine		Cimetidine	Phenytoin
Nalidixic acid			Grapefruit juice	Rifampicin
Nitrofurantoin			Erythromycin	Macrolide antibiotics
Niridazole			Ritonavir	Glucocorticoids
Aspirin				
Traditional medicines				

#### Cost-of-illness assessments

A total of two household cost questionnaires will be completed on enrolment and on day 13/14. The second one will capture all costs following the attendance on day 0, including the duration of the episode and associated productivity losses, further expenditure for consultation, medication, investigations or admissions, travel costs (other than those for research purposes).

#### Definition of end of trial

The end of trial is the date of the last visit of the last participant, which would be a maximum of 365 days after recruitment of the last participant. No participant will be followed up for longer than 365 days from enrolment regardless of repeated episode of parasitaemia.

#### Withdrawal

Patients withdrawn from the study will not have any further protocol mandated study investigations or procedures at and after the point of withdrawal. Each participant has the right to withdraw from the study at any time (withdrawal of consent).

Other reasons for study withdrawal include:

- ○ Ineligibility (either arising during the study or retrospective having been overlooked at screening)
- ○ Significant protocol violation e.g. given wrong dose of primaquine/placebo
- ○ Significant non-compliance with treatment regimen or study requirements
- ○ An adverse or serious adverse event which results in inability to continue to comply with study procedures
- ○ Disease progression which results in an inability to continue to comply with study procedures
- ○ Lost to follow up (patients fail to return for follow up right up to study day 365)

Summary information on withdrawn patients will be recorded in the final status CRF. If the participant is withdrawn from the study because of an adverse event, the investigator will arrange for follow-up visits or telephone calls until the adverse event has resolved or stabilised.

There will be instances when patients may have adverse events that result in temporary interruption of their study drugs or a break in their follow up. When these have resolved, patients will still remain in the study and be followed up according to the study schedule. Examples include:

- ○ Persistent vomiting of study drug/s
- ○ A serious adverse event which requires stopping study drug/s
- ○ Disease progression which requires discontinuation of the study medication An emergency event requiring unblinding of the drug regimen code allocated to the participant

#### Safety reporting

**Adverse Event (AE): **An AE or adverse experience is any untoward medical occurrence in a patient or clinical investigation participants administered a medicinal product, which does not necessarily have to have a causal relationship with this treatment (the study medication). An AE can therefore be any unfavourable and unintended sign (including an abnormal laboratory finding), symptom or disease temporally associated with the use of the study medication, whether or not considered related to the study medication.

**Adverse Reaction (AR):** Adverse reactions are defined as untoward and unintended responses to a medicinal product related to any dose. The phrase “responses to a medicinal product” infers that a causal relationship between a study medication and an AE is at least a reasonable possibility, i.e., the relationship cannot be ruled out. All cases judged by either the reporting medically qualified professional or the sponsor as having a reasonable suspected causal relationship to the study medication qualify as adverse reactions.

**Serious Adverse Event (SAE):** A serious adverse event is any untoward medical occurrence that results in death, is life-threatening, requires inpatient hospitalisation or prolongation of existing hospitalisation, results in persistent or significant disability/incapacity, or is a congenital anomaly/birth defect.

Other events that may not result in death are not life threatening, or do not require hospitalisation, may be considered a serious adverse event when, based upon appropriate medical judgement, the event may jeopardise the patient and may require medical or surgical intervention to prevent one of the outcomes listed above.

**Serious Adverse Reaction (SAR):** An adverse event (expected or unexpected) that is both serious and, in the opinion of the reporting investigator, believed with reasonable probability to be due to one of the study treatments, based on the information provided.

**Suspected Unexpected Serious Adverse Reaction (SUSAR):** A serious adverse reaction, the nature or severity of which is not consistent with the applicable product information (e.g. Investigator’s Brochure for an unapproved investigational product or summary of product characteristics for an approved product).

**Causality and Expectedness:** The relationship of an adverse event to study medication is to be assessed according to the following definitions:

- ○ Definitely Related: Strong evidence exists that the study drug caused the adverse event. There is a temporal relationship between the event onset and administration of the study drug. There is strong therapeutic and pharmacologic evidence that the event was caused by the study drug. The subject’s clinical state or concomitant therapies have been ruled out as a cause. In the case of cessation or reduction of the dose, the event abates or resolves, and reappears upon re-challenge.
- ○ Probably Related: A temporal relationship exists between the event onset and the administration of study drug, and appears with some degree of certainty to be related based on the known therapeutic and pharmacologic actions of the study drug. It cannot be readily explained by the subject’s clinical state or concomitant therapies. In the case of cessation or reduction of the dose, the event abates or resolves.
- ○ Possibly Related: A temporal relationship exists between the event onset and the administration of study drug. Although the adverse event may appear unlikely to be related to the study drug, it cannot be ruled out with certainty; and/or the event cannot be readily explained by the subject’s clinical state or concomitant therapies.
- ○ Not Related: Evidence exists that the adverse event definitely has aetiology other than the study drug (e.g., pre-existing condition or underlying disease, other illness, or concomitant medication) and does not meet any criteria listed above.

**Recording AEs:** All grades 3 and 4 adverse events should be recorded for up to 28 days after the last day of administration of primaquine in G6PD normal patients (i.e. Day 42) and up to 14 days after the last dose in G6PD deficient patients. All related AEs that result in a participant’s withdrawal from the study or are present at the end of the study, should be followed up until a satisfactory resolution occurs. It will be left to the Investigator’s clinical judgment whether or not an AE is of sufficient severity to require stopping the participant’s treatment. A participant may also voluntarily withdraw from treatment due to what he or she perceives as an intolerable AE. If either of these occurs, the participant must undergo an end of study assessment and be given appropriate care under medical supervision until symptoms cease or the condition becomes stable.

The severity of events will be assessed on the following scale: 1 = mild, 2 = moderate, 3 = severe, 4 = potentially life-threatening. Any pregnancy occurring during the clinical study and the outcome of the pregnancy should be recorded and followed up for congenital abnormality or birth defect.

**Reporting Procedures for Serious Adverse Events:** All SAEs will be reported by the site investigator to the MORU study coordinator and pharmacovigilance officer within one working day of awareness, who will in turn notify the DSMB and PI within 48 h of their being notified of the event. The site PI will be responsible for notifying the local Ethical Committees as appropriate. All information received for a case will be detailed on a full SAE report form.

**DMSB:** A Data Safety Monitoring Board (DSMB) will be appointed to review the study progress, safety data, critical efficacy outcomes, at regular intervals and recommend to the sponsor whether to continue, modify, or stop the trial.

**Annual Safety reports:** In addition to the expedited reporting above, the PI shall submit an annual safety report throughout the clinical trial, and any additional reports on request.

#### Management of patients with anaemia related adverse events

Malaria can cause a temporary fall in haemoglobin that can be exacerbated by primaquine (Additional file 1).

Patients may report symptoms that are suggestive of anaemia and / or acute intravascular haemolysis or may have decline in the HemoCue measured Hb that may be concerning for the attending clinician. All patients in whom the attending clinician is concerned about will be assessed, including a history, recent non study drug intake, a clinical examination, urine inspection for colour, measure the Hb and retest for G6PDd (daily arm only) with the fluorescent spot test. If any of the following are present then primaquine/placebo study drug will be stopped:

- A fall in haemoglobin concentration below 7 g/dL
- A blood transfusion
- Macroscopic haemoglobinuria (Hillmen ≥ 5) AND a fractional fall in haemoglobin ≥ 25 % from baseline

Patients reaching any of these criteria will have their primaquine/placebo stopped. They will be unblinded from the study but will continue to be followed for the remainder of their 1 year follow up. The need for admission to hospital for observation or to receive treatment including a blood transfusion is a serious adverse event (SAE) by definition.

If the patient has any of the following features primaquine will be withheld, pending close monitoring:

- A fractional Hb drop ≥ 25 % from baseline OR
- Urine colour ≥ 5 (macroscopic haemoglobinuria) OR
- Other significant clinical concern of increasing anaemia or severe haemolysis

An adverse event form must be filled for those patients with clinical concern who had their primaquine/placebo trial drug therapy withheld but later recommenced.

#### Redosing primaquine after a blood transfusion

Following a severe haemolytic reaction, patients are at significant risk of further haemolysis if dosed with primaquine again. In some settings local clinicians regard the risk of multiple recurrent episodes warrant careful rechallenge with primaquine. However the decision to redose with open label primaquine will be made by the attending local clinician. It is not a protocol requirement. The physician will assess the risks and benefits and discuss them with the patient. In patients who have had severe haemolysis, the risk of further drug induced haemolysis will be weighed against the risk of recurrent malaria causing haemolysis and anaemia.

If primaquine is prescribed then this should be at the weekly dose of 0.75 mg /kg to complete a total dose of 6 mg/kg. Redosing should be limited to those patients with no signs of ongoing haemolysis, a Hb above 9 g/dL, no anaemia related symptoms and no signs evidence of clinical compromise.

### Sample size

The primary aim of this trial is to demonstrate non-inferiority of a 7 day primaquine regimen to the standard 14 day primaquine regimen with respect to incidence rate of vivax symptomatic parasitaemia over 12 months. This will reflect mainly relapse in these relatively low transmission settings. The sample size calculation is based on an assumed incidence rate of 0.2 infections per person-year in both arms, a non-inferiority margin of 0.07 infections per person-year and a one-sided significance level of 2.5 %. Based on these assumptions, a total sample size of 1200 evaluable patients, randomly allocated to receive a primaquine regimen (600 patients in each treatment arm), followed for one year will provide a power of 80 % to show non-inferiority or, equivalently, that the two-sided 95 % confidence interval for the difference in incidence rate of malaria between the two arms excludes an excess rate of 0.07 infections per person-year or more in favour of the 14 day regimen.

A further 300 patients in the control arm will also be followed for one year. With 300 patients in the control arm and 600 patients in each treatment arm (i.e. 75 controls and 150 patients in each treatment arm at each of the five study sites), the study has 95 power and 95 % confidence to detect a difference (i.e. a superiority comparison of either regimen) at each of the study sites assuming an incidence rate of 0.2 infections per person-year in each of the treatment arms and 0.6 infections per person-year in the control arm (ranging from 0.2 in the Indian subcontinent and 1.0 in Vietnam and Indonesia). The combined proportion of losses to follow-up and major protocol violations is expected to be no more than 20 %, so to account for this, a total of 1875 G6PD normal patients (750 per treatment arm; 375 in the control arm) will be randomized in this trial. In addition, up to 380 patients (approximately 20 % of the screened participants) with G6PD deficiency will be enrolled for the non-randomised observational study.

With the addition of two extra sites, Ethiopian and northern Sumatra, we expect to exceed the original sample size calculation and, thus, have increased power.

### Statistical plan

A detailed statistical analyses plan has been developed and agreed on among the investigators (Additional file 2).

### QC and QA

The study will be conducted in accordance with the current approved protocol, ICH GCP, relevant regulations and standard operating procedures. Regular monitoring will be performed. Data will be evaluated for compliance with the protocol and accuracy in relation to source documents. Following written standard operating procedures, the monitors will verify that the clinical trial is conducted and data are generated, documented and reported in compliance with the protocol.

### Protocol changes, ethics and dissemination

#### Protocol changes

From initial submission until to date the protocol has undergone few adjustments. The most important modification are listed in table 4.

**Table 4:** List of amendment until to date

Amendment number	Protocol version no	Changes
1	V 1.1	• Amendments in response to OXTREC, HREC comments
2	V 2.1 and V 2.2	• Updated visit schedule
3	V 2.11 to V2.15	• Maximum number of symptomatic recurrences limited to 4 then supervised open PQ will be given • Primaquine also to be given supervised for 14 days at the appropriate dose to all participants with at least 1 symptomatic or asymptomatic relapse at the end of the study • Option for telephoning subjects to run though symptom checklist • Patients can be enrolled into the study on the basis of a positive RDT for malaria, as per site local practice • Amend haematological warning signs & management of acute haemolysis
4	V 2.16	• Inclusion of flow cytometry in addition to the currently proposed G6PD tests
5	V 2.16-3.0	• Removal of taking a drug with antimalarial activity during the study as reason to withdraw patient • Add exclusion criterion – cannot be enrolled in IMPROV twice • Exclusion criteria removed: women planning to become pregnant & previous use of antimalarial drugs • G6PD status to be decided on basis of spot test only • Methaemoglobin to be measured at selected sites only and rotated • Assent form amended to include weekly PQ regimen • HemoCue sampling increased in both arms • Maximum number of symptomatic recurrences in Vietnam – 3 within first 6 months – added to the consent form but will be for Vietnam only • Adjustment to dosing table for those weighing 17 kg
6	V 4.0	• Addition of Ethiopia • G6PD substudy with consent form added as an appendix • Study schedule amended accordingly to accommodate additional G6PD testing • Clarity added on PK sampling on D7 & 14 – now to be done pre & post dose
7	V 5.0	• Update list of investigator and affiliations • Remove site in Pakistan and add the 2^nd^ site in Ethiopia • Revise AE reporting to grades 3 and 4 only • Revise management of patients with anemia related adverse events & management of acute haemolysis

#### Ethics

Ethical approval for the original protocol and included amendments (Version 5) is obtained from the following review boards: Oxford Tropical Research Ethics Committee OxTREC (Ref number 1014–13) and the Human Research Ethics Committee of the Northern Territory Department of Health, Australia HREC (Ref Number 13–1991). In addition local approvals were obtained from the National Bioethics Committee (NBC) in Pakistan (this site was dropped before start of enrolment), the Institutional Review Board, Ministry of Public Health, Afghanistan, the Health Research Ethics Committee, Faculty of Medicine University of Indonesia, Cipto Mangunkusumo Hospital, Jakarta, Indonesia, the Ministry of Health Evaluation Committee on Ethics in Biomedical Research Vietnam, the Institutional Scientific & Ethical Review Committee of the Ethiopian Public Health Institute, the National Research Ethics Review Committee, Ethiopia and the Institutional Review Board of the Columbia University Medical Centre, US.

Indemnity for the trial is provided by the University of Oxford and Menzies School of Health Research. SAEs will be reported to the DSMB, ethics committees and the sponsor’s research office. Good Clinical Practice (GCP) training is provided to all staff/investigators prior to commencing the studies. Other ethical consideration can be found in Additional file 3.

#### Dissemination

All Investigators will be involved in reviewing drafts of the manuscripts, abstracts, press releases and any other publications arising from the study. Authorship will be determined in accordance with the ICMJE guidelines and other contributors will be acknowledged.

## Additional files

Additional file 1:
**Standard operating procedures (SOP) for haemolyses, SOP version 2.0, dated 26 June 2015.** (PDF 960 kb) Additional file 2:
**Statistical Analytical Plan (SAP), version number 1.0.** (PDF 330 kb) Additional file 3:
**Ethical considerations for the IMPROV study.** (PDF 241 kb)
