# Supplementary material for: Improving the radical cure of vivax malaria (IMPROV): a study protocol for a multicentre randomised, placebo-controlled comparison of short and long course primaquine regimens
Source: BMC Infect Dis. 2015 Dec 7;15:558. doi: 10.1186/s12879-015-1276-2 (PMC4672497; doi:10.1186/s12879-015-1276-2)
Supplement: Additional file 1: — Standard operating procedures (SOP) for haemolyses, SOP version 2.0, dated 26 June 2015. (PDF 960 kb) [file 12879_2015_1276_MOESM1_ESM.pdf]

# Statistical Analysis Plan

## Improving the radical cure of vivax malaria

## (IMPROV)

**Multicentre randomized comparison of short and long course primaquine regimens**

**Version number: 1.0**

**Date: 17th June 2015**

## Contents

1. Introduction ... 5
2. Study design and objectives ... 5
   2.1. Summary of trial design ... 5
   2.2. Objectives ... 6
      Primary Objective ... 6
      Secondary Objectives ... 6
   2.3. Sample size ... 6
**3. Endpoints and Definitions** ... 7
   3.1. Endpoints ... 7
      3.1.1. Efficacy Endpoints ... 7
      3.1.1.1. Primary efficacy endpoints (Radical Cure) ... 7
      3.1.1.2. Secondary efficacy endpoints (Radical Cure) ... 7
      3.1.2. Safety Endpoints ... 10
   3.2. Definitions ... 11
      3.2.1. Symptomatic patients ... 11
      3.2.2. Study site ... 11
   3.3. Handling of missing data and adjudication of Endpoints ... 11
      3.3.1. Handling of missing data on drug course ... 11
      3.3.2. Handling of missing data for Adverse Events ... 11
      3.3.3. Adjudication of day 42-365 efficacy outcome assessment ... 11
      3.3.4. Adjudication of day 28 and 42 efficacy outcome assessment ... 12
      3.3.5. Adjudication for endpoint on parasite prevalence on day 0-3 ... 12
      3.3.6. Adjudication for endpoint on fever clearance on day 0-3 ... 12
      3.3.7. Adjudication for haemoglobin outcome ... 12
   3.4. Analyses Populations ... 13
      3.4.1. Efficacy population ... 13
      3.4.2. Safety population ... 14
      3.4.3. Pharmacokinetic population ... 14
4. Analyses ... 14
   4.1. General analyses strategy ... 14
   4.2. Pooling across sites ... 14
   4.3. Assessment of heterogeneity ... 14
   4.4. Demographic and baseline characteristics ... 14
      4.4.1. Demographic characteristics ... 15

4.4.2. Disease characteristics at baseline ..... 15
4.4.3. Prior or concomitant medications ..... 15
4.5. Efficacy analyses ..... 15
4.5.1. *P. vivax* radical cure ..... 15
4.5.2. *P. vivax* schizontocidal efficacy ..... 17
4.5.3. *P. falciparum* schizontocidal efficacy ..... 17
4.6. Primary efficacy analyses (Radical Cure) ..... 17
4.6.1. Incidence rate of symptomatic *P. vivax* parasitaemia over 12 months ..... 17
4.7. Secondary efficacy analyses (Radical Cure) ..... 18
4.7.1. Incidence rate of symptomatic P. vivax parasitaemia over 12 months ..... 18
4.7.2. Incidence rate of any *P. vivax* parasitaemia over 12 months ..... 18
4.7.3. Incidence risk of symptomatic recurrence of *P. vivax* over 12 months ..... 18
4.7.4. Incidence risk of any recurrence of *P. vivax* over 12 months ..... 19
4.7.5. Incidence rate of any symptomatic parasitaemia over 12 months ..... 19
4.7.6. Incidence rate of any parasitaemia over 12 months ..... 19
4.7.7. Incidence risk of any symptomatic parasitaemia over 12 months ..... 19
4.7.8. Incidence risk of any parasitaemia over 12 months ..... 19
4.8. Secondary efficacy analyses (Schizontocidal Efficacy) ..... 20
4.8.1. Schizontocidal efficacy of chloroquine and chloroquine plus primaquine at day 28 and 42 20
4.8.2. PCR-adjusted incidence risk of *P. vivax* at day 28 and 42 ..... 20
4.8.3. Proportion of patients with *P. vivax* parasitaemia on day 1, 2 and 3 after schizontocidal treatment in the control arm ..... 20
4.8.4. Proportion of patients with fever on day 1, 2 and 3 after schizontocidal treatment for *P. vivax* in the control arm ..... 20
4.8.5. Proportion of patients with *P. falciparum* parasitaemia on day 1, 2 and 3 after treatment with ACT during follow up ..... 20
4.8.6. Proportion of patients with fever on day 1, 2 and 3 after schizontocidal treatment for *P .falciparum* during follow up ..... 21
4.9. Additional analyses: ..... 21
4.9.1. Incidence risk of *P. falciparum* after *P. vivax* infection ..... 21
4.9.2. Incidence risk of *P. vivax* after first *P. falciparum* infection ..... 21
4.9.3. The distribution of *P. vivax* recurrences in the control arm ..... 21
4.9.4. Subgroup analyses ..... 21
4.10. Safety analyses ..... 22
4.10.1. Risk of Haemolyses ..... 22

4.10.2. Risk of Adverse Events ........ 23
4.10.2.2. Risk of gastroenterological events ........ 23
4.10.2.3. Overall risk of Adverse Events (AE) and Serious Adverse Events (SAE) ........ 23
4.11. Pharmacokinetic analyses ........ 23
4.11.1. Chloroquine ........ 23
4.11.2. Primaquine ........ 24

# 1. Introduction

This Statistical Analysis Plan (SAP) provides a detailed and comprehensive description of the main pre-planned analyses for the clinical trial "Improving the radical cure of *P. vivax* malaria (IMPROV): A multicentre randomised, placebo-controlled comparison of short and long course primaquine regimens".

The main purpose of the trial is to determine whether a 7-day primaquine regimen is safe and non inferior to the standard 14-day regimen (total dose of 7mg/kg in both arms) in preventing *P. vivax* relapse in G6PD normal patients. The study conduct is described in Protocol OXTREC 1014-13 and registered with ClinicalTrials.gov under NCT01814683.

This document describes the statistical methods for the primary and secondary outcomes of the study as defined by the protocol as well as additional analyses of drug concentration data and a brief outline of further analyses in regards to parasite genetic studies, host genotyping, quantitative analysis of G6PD, evaluation of novel G6PD test and serology. The analysis plan for the economic component of the study will be presented separately.

# 2. Study design and objectives

## 2.1. Summary of trial design

This is a randomized, double-blind, placebo-controlled, non-inferiority trial in G6PD normal patients with uncomplicated vivax malaria in the five participating study sites. Patients presenting to a participating treatment centre with uncomplicated vivax malaria and fulfilling the enrolment criteria will be randomly assigned to one of three treatment arms:

- **Intervention 1**: Standard blood schizonticidal therapy plus 14 days of supervised primaquine (7mg/kg total dose) administered once per day (0.5 mg/kg).
- **Intervention 2**: Standard blood schizonticidal therapy plus 7 days of supervised primaquine (7mg/kg total dose) administered once per day (1.0 mg/kg OD) followed by 7 days of placebo.
- **Control arm**: Standard blood schizonticidal therapy plus 14 days placebo.

Patients tested initially and found to be G6PD deficient will be excluded from the randomized study, but offered enrolment in a single arm non-randomised observational study to receive standard blood schizontocidal therapy plus primaquine 0.75 mg/kg/week for 8 weeks (total dose 6 mg/kg).

All patients will receive standard medical care for the management of uncomplicated malaria, with blood schizontocidal treatment administered as either chloroquine (total dose 25 mg base/kg) or an artemisinin combination therapy such as a three-dose regimen of dihydroartemisinin-piperaquine, depending on local recommendations and known chloroquine efficacy. Recurrences of any species within 28 days will be considered treatment failures and treated with local second line alternatives (such as ACT or 7 days quinine) according to national guidelines. After 28 days, treatment failure is

less likely and so patients will be treated with the same treatment regimen as that allocated at enrolment.

Primaquine/placebo will be administered with food (crackers or a biscuit), which has been shown to reduce gastrointestinal side effects. All doses of study drugs will be supervised. If participants cannot visit the study centre, or fail to attend during the 14 days of supervised therapy, team members will visit them in their homes or places of school or work to ensure complete dosing. Treatment efficacy and patient safety will be ensured by close monitoring over a 12 month follow up period following a schedule of visits and corresponding clinical and laboratory examinations. Individuals will be reviewed regularly and sampled for haemoglobin and blood film examination, the latter will be read immediately if the patient is symptomatic/febrile, or in a reference centre at a later date if the patient is well. Each symptomatic recurrence of *P. vivax* will be confirmed by microscopy and recorded. Intercurrent *P. falciparum* parasitaemia will be treated with the locally recommended ACT.

## 2.2. Objectives

**Primary Objective**

- To determine whether a 7-day primaquine regimen is safe and non inferior to the standard 14-day regimen (total dose of 7mg/kg in both arms) in preventing *P. vivax* relapse in G6PD normal patients.

**Secondary Objectives**

- To assess the absolute risks and benefits of radical treatment regimens in different endemic settings.
- To provide data on the safety of a weekly dose of primaquine (0.75 mg base/kg) in patients with G6PD deficiency.
- To identify the most cost-effective strategies for the management of *P. vivax* with respect to the use of G6PD tests, the dosing schedule and the epidemiological context (not covered in this SAP).

## 2.3. Sample size

The sample size calculation is based on an assumed incidence rate of 0.2 vivax infections per person-year in both arms, a non-inferiority margin of 0.07 infections per person-year and a one-sided significance level of 2.5%. Based on these assumptions, a total sample size of 1200 evaluable patients, randomly allocated to receive a primaquine regimen (600 patients in each treatment arm), followed for one year will provide a power of 80% to show non-inferiority or, equivalently, that the two-sided 95% confidence interval for the difference in incidence rate of malaria between the two arms excludes an excess rate of 0.07 infections per person-year or more in favour of the 14 day regimen. A further 300 patients in the control arm will also be followed for one year. With 300 patients in the control arm and 600 patients in each treatment arm (i.e. 75 controls and 150 patients in each treatment arm at each of the five study sites), the study has 95% power and 95% confidence to detect a difference (i.e. a superiority comparison of either regimen) at each of the study sites assuming an incidence rate of 0.2 infections per person-year in each of the treatment arms and 0.6 infections per person-year in the control arm (ranging from 0.2 in the Indian subcontinent and 1.0 in Vietnam and Indonesia). The combined proportion of losses to follow-up and major protocol

violations is expected to be no more than 20%, so to account for this, a total of 1875 G6PD normal patients (750 per treatment arm; 375 in the control arm) will be randomized in this trial. In addition, up to 380 patients (approximately20% of the screened participants) with G6PD deficiency will be enrolled for the non-randomised observational study.

# 3. Endpoints and Definitions

## 3.1. Endpoints

### 3.1.1. Efficacy Endpoints

All efficacy endpoints refer to risk of recurrent parasitaemia which, unless otherwise specified, are based on microscopy (table 1 provides an overview).

#### 3.1.1.1. Primary efficacy endpoints (Radical Cure)

**3.1.1.1.1.** Incidence **rate** (per person year) of **symptomatic** recurrent ***P. vivax*** **parasitaemia** over 12 months of follow-up, for all individuals, controlling for site, comparing the 7 versus 14-day primaquine treatment arms.

#### 3.1.1.2. Secondary efficacy endpoints (Radical Cure)

**3.1.1.2.1.** Incidence **rate** (per person year) of **symptomatic** recurrent ***P. vivax*** **parasitaemia** over 12 months follow-up, for all individuals, controlling for site, comparing the 7 and the 14-day primaquine treatment arms with the control arm.

**3.1.1.2.2.** The incidence **rate** (per person-year) of **any** recurrent (i.e. **symptomatic and asymptomatic**) ***P. vivax*** **parasitaemia** over 12 months of follow-up, for all individuals, controlling for site and comparing the 7 day with the 14- day primaquine regimen and the control arm with each primaquine regimen.

**3.1.1.2.3.** The incidence **risk** (*note, from hereafter in this document the incidence risk refers to the conditional probability of recurrence at time t, given the individual has not had a recurrence prior to this time, calculated using the Kaplan-Meier method*) of **symptomatic recurrence of *P. vivax*** over 12 months of follow up, for all individuals, controlling for site, comparing the 7 day with the 14- day primaquine regimen and the control arm with each primaquine regimen.

**3.1.1.2.4.** The incidence **risk** of **any (symptomatic and asymptomatic) recurrence of *P. vivax*** over 12 months of follow up, for all individuals, controlling for site comparing the 7 day with the 14- day primaquine regimen and the control arm with each primaquine regimen.

**3.1.1.2.5.** The incidence **rate** of **any symptomatic parasitaemia (all species)** over 12 months of follow-up, for all individuals, controlling for site, comparing the 7 day with the 14- day primaquine regimen and the control arm with each primaquine regimen.

**3.1.1.2.6.** Incidence rate of **any (symptomatic and asymptomatic) parasitaemia (all species)** over 12 months of follow-up, for all individuals, controlling for site, comparing the 7 day with the 14- day primaquine regimen and the control arm with each primaquine regimen.

**3.1.1.2.7.** The incidence **risk** of **any symptomatic parasitaemia (all species)** over 12 months of follow up, for all individuals, controlling for site, comparing the 7 day with the 14- day primaquine regimen and the control arm with each primaquine regimen.

**3.1.1.2.8.** The incidence **risk** of **any (symptomatic and asymptomatic) parasitaemia (all species)** over 12 months of follow up, for all individuals, controlling for site, comparing the 7 day with the 14- day primaquine regimen and the control arm with each primaquine regimen.

#### 3.1.1.3. Secondary Efficacy endpoints (Schizontocidal Efficacy)

All secondary efficacy endpoints refer to the treatment outcome following the first episode of *P. vivax* infection (enrolment episode) or the first symptomatic episode of *P. falciparum*.

**3.1.1.3.1.** The incidence **risk** of *P. vivax* recurrence of the schizontocidal therapy at day 28 and 42 in the control arm and in the intervention arms.

**3.1.1.3.2.** PCR-adjusted incidence **risk** of *P. vivax* at day 28 and 42 in the control arms and in the intervention arms.

**3.1.1.3.3.** Proportion of patients with *P. vivax* parasitaemia on days 1, 2 and 3 after schizontocidal treatment in the control arm.

**3.1.1.3.4.** Proportion of patients with fever on days 1, 2 and 3 after schizontocidal treatment for *P. vivax* in the control arm.

**3.1.1.3.5.** Proportion of patients with *P. falciparum* parasitaemia on days 1, 2 and 3 after treatment of *P. falciparum* recurrent parasitaemia with ACT.

**Table 1: Primary and secondary efficacy endpoints (incidence risk and rate for radical cure and schizontocidal efficacy)**

| **Endpoint** | **Number** | **Risk/Rate** | **Type of recurrence** | **Duration of follow up, days** | **Species of recurrence** | **Comparison** |
|---|---|---|---|---|---|---|
| Primary | 3.1.1.1.1 | Rate | Symptomatic | *365* | *P. vivax* | 7 vs 14 |
| Secondary | 3.1.1.2.1 | Rate | Symptomatic | *365* | *P. vivax* | 7 vs control; 14 vs control |
| Secondary | 3.1.1.2.2 | Rate | Symptomatic & asymptomatic | *365* | *P. vivax* | 7 vs 14; 7 vs control; 14 vs control |
| Secondary | 3.1.1.2.3 | Risk | Symptomatic | *365* | *P. vivax* | 7 vs 14; 7 vs control; 14 vs control |
| Secondary | 3.1.1.2.4 | Risk | Symptomatic & asymptomatic | *365* | *P. vivax* | 7 vs 14; 7 vs control; 14 vs control |
| Secondary | 3.1.1.2.5 | Rate | Symptomatic | *365* | All species | 7 vs 14; 7 vs control; 14 vs control |
| Secondary | 3.1.1.2.6 | Rate | Symptomatic & asymptomatic | *365* | All species | 7 vs 14; 7 vs control; 14 vs control |
| Secondary | 3.1.1.2.7 | Risk | Symptomatic | *365* | All Species | 7 vs 14; 7 vs control; 14 vs control |
| Secondary | 3.1.1.2.8 | Risk | Symptomatic & asymptomatic | *365* | All Species | 7 vs 14; 7 vs control; 14 vs control |
| Secondary | 3.1.1.3.1 | Risk | Symptomatic & asymptomatic | *28/42* | *P. vivax* | 7 vs 14; 7 vs control; 14 vs control |
| Secondary | 3.1.1.3.2 | Risk | Symptomatic & asymptomatic, PCR adjusted | *28/42* | *P. vivax* | 7 vs 14; 7 vs control; 14 vs control |

### 3.1.2. Safety Endpoints

#### 3.1.2.1. Risk of Haemolysis

**3.1.2.1.1.** Incidence risk of **severe anaemia** (Hb<7g/dl) and/or the risk for blood transfusion within the 12 month follow up period.

**3.1.2.1.2.** Incidence risk of an acute **drop in Hb >5g/dl** within 7 days of starting of primaquine treatment.

**3.1.2.1.3.** **Hb concentration** on day 3 and day 7.

**3.1.2.1.4.** **Hb nadir** (defined as minimum Hb measurement during 28 days following treatment) and the median time at which it occurs.

**3.1.2.1.5.** **Area under the curve** for day 0-28, 28-365 and 0-365, assuming the Hb value of day 28 to be the normal value (see Figure 1 for example).

**Figure 1: Area under the curve**

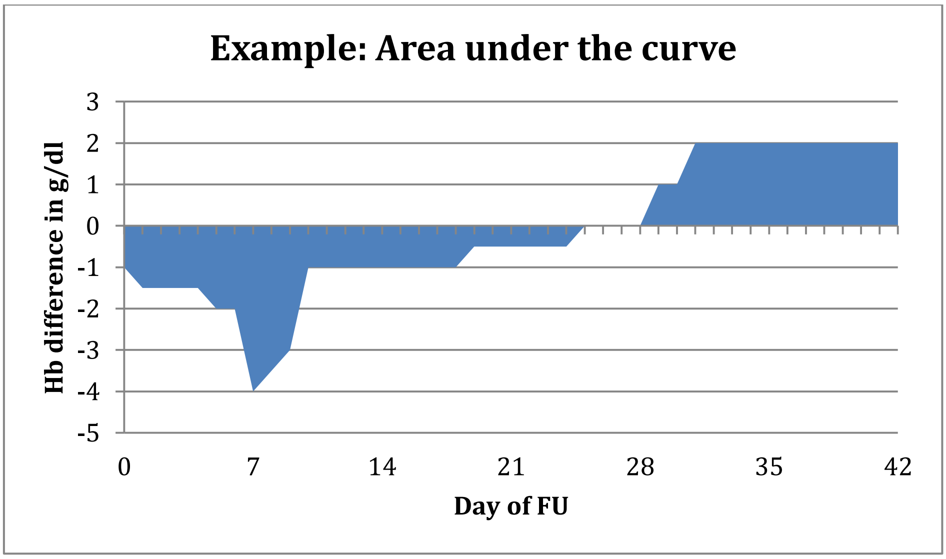


#### 3.1.2.2. Risk of adverse events

**3.1.2.2.1.** Proportion of patients with **vomiting within 1 hour of administration** of a dose comparing the intervention arms and each intervention arm with the control arm.

**3.1.2.2.2.** Proportion of patients with **nausea, vomiting or abdominal pain** comparing the intervention arms and each intervention arm with the control arm.

**3.1.2.2.3.** The **incidence risk of one or more adverse events and serious adverse events** within 7, 14 and 42 days of the primary treatment comparing the intervention arms and each intervention arm with the control arm.

**3.1.2.2.4.** The incidence rate of serious adverse event and serious adverse events over the study period comparing the intervention arms and each intervention arm with the control arm

**3.1.2.2.5.** Proportion of patients completing a **full course** of all observed and blinded primaquine therapy comparing the intervention arms and each intervention arm with the control arm.

## 3.2. Definitions

### 3.2.1. Symptomatic patients

For the purpose of establishing the primary efficacy endpoints, “symptomatic patients” are defined as patients with *P. vivax* parasitaemia and either a documented fever (axillary temperature ≥37.5°C) or a history of fever within the preceding 48 hours.

### 3.2.2. Study site

A study site is defined as study location(s) under the responsibility of one local Principal investigator (PI) (e.g. one site could consist of two study hospitals in the same area under the responsibility of the same PI).

## 3.3. Handling of missing data and adjudication of Endpoints

### 3.3.1. Handling of missing data on drug course

Patients can have an incomplete course of treatment or data on drug administration may be missing. No imputation of treatment course will be made for patients with missing data. Subgroup analyses will be performed for patients with full versus partial treatment courses (see section 4.9.3).

### 3.3.2. Handling of missing data for Adverse Events

For patients with missing data on adverse events the most conservative approach will be used:

| Deviation | Action |
|---|---|
| Start date of AE missing | Assume during study drug intake |
| End date of AE missing | No imputation |
| Date of start of study treatment administration missing | All AEs after randomization considered to have happened during study drug intake |
| Missing assessment of relationship to study treatment | Assume event to be possibly related |
| Missing severity assessment of AE | Assume highest severity |

### 3.3.3. Adjudication of day 42-365 efficacy outcome assessment

For missed visits during the 1 year follow up, the following rules will apply:

| Deviation | Action |
|---|---|
| More than 2 consecutive months without blood film examination | -In the survival analysis lost to follow up (i.e. censored) on day of last visit before missing observation period.<br>-In the incidence rate analysis using asymptomatic recurrences as the event, the period of missing observation will be deducted from the total period of observation.<br>-In the incidence rate analysis using symptomatic recurrences as the event, the |

| | |
|---|---|
| | period of missing observation will not be deducted from the total period of observation. |

### 3.3.4. Adjudication of day 28 and 42 efficacy outcome assessment

The clinical definition of treatment failure enables determination of the efficacy endpoint for schizontocidal activity. However when the number of scheduled visits is incomplete the following rules will be applied[1]:

| Outcome assessment | Deviation | Action |
|---|---|---|
| **Day 28 and 42** | More than 18 days without blood smear results | Lost to follow up on the day of last observation |
| **Day 28** | No blood smear results between day 25 and day 31 | Lost to follow up on day of last visit before day 25 |
| **Day 42** | No blood smear results between day 39 and day 45 | Lost to follow up on day of last visit before day 39 |

### 3.3.5. Adjudication for endpoint on parasite prevalence on day 0-3

For missed blood films (microscopy) the following rules will be applied1:

| Parasitaemia by microscopy | | | | Action |
|---|---|---|---|---|
| **Day 0** | **Day 1** | **Day 2** | **Day 3** | |
| Yes | Yes | No | Missing | Assumed no parasitaemia on day 3 |
| Yes | Yes | Missing | Yes | Assumed parasitaemia on day 2 |
| Yes | No | Missing | Missing | Assumed no parasitaemia on day 2 and 3 |
| Yes | Yes | Missing | No | Will not contribute to proportion of patients with parasitaemia on day 2 |
| Yes | No | Missing | No | Assumed no parasitaemia on day 2 |
| Yes | No | Missing | Yes | Will not contribute to proportion of day 2 with parasitaemia |
| Yes | Yes | Missing | Missing | Will not contribute to proportion of patients with parasitaemia on day 2 or 3 |

### 3.3.6. Adjudication for endpoint on fever clearance on day 0-3

For missed temperature records, the same rules as for missing blood slides will apply.

### 3.3.7. Adjudication for haemoglobin outcome

For missing haemoglobin measurements during follow up the following rules apply:

| Outcome assessment | Deviation | Action |
|---|---|---|
| Incidence risk of severe anaemia within 12 months | 2 or more consecutive visits (only visits with scheduled Hb measurement) without Hb measurement | lost to follow up on day of last visit before missing observation period |

[1] WWARN. Clinical Module, Data Management and Statistical Analyses Plan. 2013; Available from: http://www.wwarn.org/sites/default/files/ClinicalDMSAP.pdf

| | | |
|---|---|---|
| Incidence risk of Hb drop within 7 days after PQ treatment | Missing Hb measurement on day 3 | Censored on day 0 |
| | Missing Hb measurement on day 7, but present on day 3 | Censored on day 3 |
| Hb level on day 3 | Missing Hb measurement on day 3 | Exclude from analysis |
| Hb level on day 7 | Missing Hb measurement on day 7 | Exclude from analysis |
| Hb nadir (during 28 days FU) | 2 or more consecutive scheduled visits within 28 days without Hb measurement | Excluded from analysis |
| Area under the curve | One scheduled visit without Hb measurement | Interpolate Hb from the two neighbouring values |
| | More than one missing scheduled visit without Hb measurement | Exclude from analysis |

## 3.4. Analyses Populations

### 3.4.1. Efficacy population

For the efficacy analysis, both an intention-to-treat (ITT) and a per-protocol (PP) approach will be adopted. The ITT analysis will be the primary approach for comparison of the different drug treatments.

#### 3.4.1.1. Intention to Treat

To provide a pragmatic comparison of the different drug treatments, the principle of intention-to-treat, will be the main strategy of analysis adopted for the primary and secondary endpoints. These analyses will be conducted on all patients assigned to the treatment groups as randomized, regardless of the study treatment received.

#### 3.4.1.2. Per Protocol

An analysis based on a "per protocol" approach will be conducted. The following will lead to exclusion in the per protocol analyses:

- Ineligibility (either arising during the study or retrospectively having been overlooked at screening)
- Significant protocol deviation e.g. given wrong total dose of primaquine/placebo

#### 3.4.2. Safety population

For the analysis of safety outcomes, all patients who received any study drug are included in the safety analysis in the treatment group they actually received. This means also patients having vomited the first dose and having had their treatment discontinued will be included in this analysis.

#### 3.4.3. Pharmacokinetic population

For the analyses of drug concentrations all patients who had at least one blood sample taken and measured will be included in the analysis, even if the measure was below the limit of quantification.

# 4. Analyses

## 4.1. General analyses strategy

The primary analysis will use the combined data from all sites and countries together, with adjustment for site effects. In addition, treatment effects will be estimated within each site, but formal assessment of non-inferiority will not be performed for each site, since the study is not powered for this comparison.

## 4.2. Pooling across sites

As this is a randomized trial, with a common protocol and data collection and review tools across all sites, this pooled approach is expected to be valid. However, evidence for heterogeneity will be visually assessed and tested before deciding whether pooling of data across sites is justified.

## 4.3. Assessment of heterogeneity

There is an expected degree of heterogeneity of exposure in different endemic settings, which might lead to heterogeneity between study sites (as defined in section 3.2.2) regarding the treatment effect. Heterogeneity between study sites will be assessed visually using Forrest plots of the site specific differences in incidence rates of vivax recurrence. I-squared values (percentage of total variation across studies due to heterogeneity) will also be calculated to assess statistically the degree of heterogeneity.

If substantial levels of heterogeneity are detected, site specific analyses will be performed only. If no substantial levels of heterogeneity are detected, then pooled analyses will be performed.

## 4.4. Demographic and baseline characteristics

Details of all patients screened, those who meet the study inclusion criteria, those who are eligible and randomized, those who are eligible but not randomized, those who withdraw from the study after randomization and those who are lost to follow-up will be summarized in a CONSORT flow diagram.

The number of patients discontinuing from the study will be tabulated by reason for study discontinuation. The number (%) of patients attending scheduled follow-up visits by study day (days 1 to 28 and 42, monthly visits until 1 year) will be reported.

The baseline value is defined as the last available value before randomization.

#### 4.4.1. Demographic characteristics

- Gender: male / female
- Median Age (years), $25^{th}$-$75^{th}$ percentiles, and range
- Age in classes: 12 months up to 5 years, 5 years up to 15 years, ≥15 years
- Median Weight (kg), $25^{th}$-$75^{th}$ percentiles, and range
- Weight in classes: <9kg / 9-<18 kg/ 18 - <36 kg / ≥ 36 kg

#### 4.4.2. Disease characteristics at baseline

Specific disease history will include the parasite and gametocytes densities (/µL) at day 0, as well as axillary temperature (°C) (quantitative and if <37.5°C), Median Pulse rate (beats/min), 25th-75th percentiles, and range and signs and symptoms (weakness, headache, anorexia, nausea, vomiting, pain, diarrhoea, convulsion, dehydration, icterus, sweat, chills, skin disorders) at day 0.

#### 4.4.3. Prior or concomitant medications

All medications taken within 28 days before randomization and until the end of the study will be reported in the case report form pages.

All medications will be coded using the World Health Organization-Drug Dictionary (WHO-DD) or equivalent dictionaries.

- Prior medications are those the patient used prior (28 days before) to first study drug intake. Prior medications can be discontinued before first administration or can be ongoing during treatment phase.
- Concomitant medications are those used during the treatment with the study drug and during the first phase of follow up until day 42. Concomitant medications can be ongoing prior medication and/or continue into post treatment medication.
- Post treatment medications are those the patient took in the period from day 43 until the end of the study.

### 4.5. Efficacy analyses

Effective treatment with schizontocidal and hypnozontocial activity is referred to as radical cure. The efficacy is confounded by exposure to reinfection. The primary analysis will focus on the overall efficacy of radical cure (see 4.5.1). The secondary analyses will investigate the subcomponents of schizontocidal efficacy (see 4.5.2).

#### 4.5.1. *P. vivax* radical cure

In the course of the follow up patients will only be tested for malaria if they have concomitant fever or a history of fever. If the blood film is positive for any blood stage Plasmodium, schizontocidal treatment will be initiated. If *P. vivax* is detected ≥28 days after initial enrolment, the patient will also receive primaquine treatment in the same regimen the patient was allocated to at enrolment.

There will be a maximum of four repeated *P. vivax* episodes treated with the originally assigned primaquine regimen (three in the case of Vietnam). Patients with their fifth or subsequent episode will be treated with open primaquine at a dose of 0.5mg/kg x 14 days and will be censored

thereafter from the primary outcome, but remain in the study for further follow up and documentation of secondary outcomes.

#### 4.5.1.1. Incidence rates

The overall primary endpoint for radical cure will be the incidence rate of recurrent episodes of symptomatic *P. vivax* parasitaemia (alone or mixed species infections). Incidence rates will be calculated by dividing the number of symptomatic *P. vivax* episodes by the number of person-years of observation (PYO) in the study population. The start date for PYO will be the day of enrolment into the study and the stop date the last visit performed (either completed study at 1 year or any last visit before lost to follow up and/or censoring). The period between start and stop dates for each patient will be calculated in days and divided by 365 to determine PYO, which will then be totalled for all participants.

For the primary endpoint comparison the absolute difference in the incidence rates between the treatment arms will be presented. Comparison between secondary incidence rate endpoints will be based on the relative difference and will therefore be presented as Incidence Rate Ratio (IRR) derived from a negative binomial regression model.

The effect of post-treatment prophylaxis on the time of observation will be assessed as follows in three sensitivity analyses:

- (i) Patients receiving antimalarial treatment during follow up will be assumed to have a period of 28 days of post- treatment prophylaxis in the case where they received chloroquine (CQ) or Dihydroartemisinin-piperaquine (DHA-PIP) and 14 days in the case where they received artemether-lumefantrine (AL). This period will thus be subtracted from their total period of follow up. In case patients receiving rescue treatment with quinine a total of 14 days will be subtracted.
- (ii) Patients receiving antimalarial treatment during follow up will be assumed to have a period of 14 days of post-treatment prophylaxis independent of the treatment received. This period will thus be subtracted from their total period of follow up.
- (iii) Patients receiving antimalarial treatment during follow up will be assumed to have no period of post-treatment prophylaxis and no time period will therefore be subtracted from their total period of follow up.

All routine slides including those from asymptomatic individuals will be read at a later date. A secondary measure of hypnozontocidal activity will therefore be the total number of blood film examinations documenting parasitaemia 14 days or more after antimalarial treatment. This secondary incidence rate of symptomatic and asymptomatic parasitaemia will be presented.

All sites except one will provide the same treatment as originally randomized for up to 4 repeated episodes of *P. vivax* during follow up. The site in Vietnam will only provide the same treatment until the 3rd episode and thereafter treat patients with open primaquine for the subsequent episodes. For the overall incidence analyses two sensitivity analyses will therefore be performed:

i) Overall, excluding the data from Vietnam
ii) Overall, including the data from Vietnam but censoring all patients at the time point of the third episode in the other countries.

#### 4.5.1.2. Incidence risk

The incidence risk (95% CI) of parasite recurrence within 12 months follow-up will be calculated using the Kaplan-Meier (KM) method for each trial arm as well as a comparison of the relative hazards between trial arms (Hazard Ratio (95% CI)) estimated from a Cox regression analysis for the time to the first recurrent episode with stratification for study site. Patients who are categorized as lost to follow up (as defined in section 3.3.3) will be censored at the day of the last visit.

Separate analyses will be performed for the incidence risk of any symptomatic *P. vivax* parasitaemia, for symptomatic and asymptomatic episodes of *P. vivax* and for any *Plasmodium* parasitaemia.

### 4.5.2. *P. vivax* schizontocidal efficacy

Schizontocidal antimalarial efficacy will be documented at day 28 and day 42. Although the primary episode at enrolment to the study will be *P. vivax*, subsequent episodes will also include non *P. vivax* species. Schizontocidal efficacy for *P. vivax* will be estimated only for the enrolment episode.

Detection of non-vivax parasitaemia before day 28 or 42 of follow up will result in antimalarial treatment, and thus censoring of the patient for the schizontocidal efficacy analyses (secondary endpoint). In the case of parasitaemia without *P. vivax* (i.e. *P. falciparum* or *P. malariae*) the individual will be considered as censored on the first day of parasitaemia. If *P. vivax* is observed either alone or mixed with other species then the individual will be considered as a treatment failure. Patients who are categorized as lost to follow up (as defined in section 3.3.4 and 3.3.3) will be censored at the day of the last visit.

The incidence risk of recurrent parasitaemia will be calculated as described in section 4.5.1.2 at day 28 and 42 instead of 12 months.

Additional measures for schizontocidal efficacy are parasite clearance time (PCT) and fever clearance time (FCT), which are not confounded by potential relapses[2]. Delayed clearance has been found to be a predictor for recurrence by day 28 and 42. Parasite prevalence on days 1, 2 and 3 will therefore be presented.

### 4.5.3. *P. falciparum* schizontocidal efficacy

Schizontocidal efficacy estimates for *P. falciparum* will only be estimated for the first <u>symptomatic</u> *P. falciparum* episode after enrolment.

Detection of non-falciparum parasitaemia before day 28 or 42 of follow up will result in antimalarial treatment, and thus censoring of the patient for the schizontocidal efficacy analyses (secondary endpoint). In the case of parasitaemia without *P. falciparum* (i.e. *P. vivax* or *P. malariae*) the individual will be considered as censored on the first day of parasitaemia.

## 4.6. Primary efficacy analyses (Radical Cure)

### 4.6.1. Incidence rate of symptomatic *P. vivax* parasitaemia over 12 months

The primary analysis for the incidence rate of **symptomatic** *P. vivax* parasitaemia over 12 months will be calculated as described in section 4.5.1.1. The 7-day treatment will be considered non-inferior to the 14-day treatment if the upper limit of the two-sided 95% confidence interval is lower

---

[2] Global extent of chloroquine-resistant Plasmodium vivax: a systematic review and meta-analysis. Price R *et al*. Lancet Infect Dis. 2014 Oct;14(10):982-91.

than 0.07 *P. vivax* infections per person-year. The analyses will be performed in the ITT and the PP population (see 3.4.1) and as follows:

- for all sites combined, controlling for site
    - excluding Vietnam
    - including the data from Vietnam, but censoring all patients at the time point of the third episode in the other countries

## 4.7. Secondary efficacy analyses (Radical Cure)

### 4.7.1. Incidence rate of symptomatic P. vivax parasitaemia over 12 months

The analysis for the incidence rate of symptomatic *P. vivax* parasitaemia over 12 months will be calculated as described in section 4.5.1.1. The following comparisons will be performed:

- 7 day regimen versus control
- 14 day regimen versus control

The analyses will be performed in the ITT and the PP population (see 3.4.1) and as follows:

- for all sites combined, controlling for site
    - excluding Vietnam
    - including the data from Vietnam, but censoring all patients at the time point of the third episode in the other countries

### 4.7.2. Incidence rate of any *P. vivax* parasitaemia over 12 months

The analysis for the incidence rate of **symptomatic & asymptomatic** *P. vivax* parasitaemia over 12 months will be calculated as described in section 4.5.1.1. The following comparisons will be performed:

- 7 days versus 14 day regimen.
- 7 day regimen versus control
- 14 day regimen versus control

The analyses will be performed in the ITT and the PP population (see 3.4.1) and as follows:

- for all sites combined, controlling for site
    - excluding Vietnam
    - including the data from Vietnam, but censoring all patients at the time point of the third episode in the other countries

### 4.7.3. Incidence risk of symptomatic recurrence of *P. vivax* over 12 months

This analysis will be performed as described in section 4.5.1.2. The first recurrent **symptomatic** episode of *P. vivax* will be treated as failure endpoint. Patients presenting with symptomatic parasitaemia other than *P. vivax* will be censored at the day or occurrence. Patients with asymptomatic parasitaemia will be censored at the time of occurrence.

Comparisons will be made between the three regimens. The analyses will be performed in the ITT and the PP population (see 3.4.1) for all sites combined, controlling for site.

#### 4.7.4. Incidence risk of any recurrence of *P. vivax* over 12 months

This analysis will be performed as described in section 4.5.1.2. The first recurrent **symptomatic or asymptomatic** episode of *P. vivax* will be treated as failure endpoint. Patients presenting with parasitaemia (symptomatic & asymptomatic) other than *P. vivax* will be censored at the day or occurrence.

Comparisons will be made between the three regimens. The analyses will be performed in the ITT and the PP population (see 3.4.1) for all sites combined controlling for site.

#### 4.7.5. Incidence rate of any symptomatic parasitaemia over 12 months

The analysis for the incidence rate of any **symptomatic** parasitaemia (*P. vivax* & any other Plasmodia) over 12 months will be calculated as described in section 4.5.1.1. The following comparisons will be performed:

- 7 day regimen versus control
- 14 day regimen versus control

The analyses will be performed in the ITT and the PP population (see 3.4.1) and as follows:

- for all sites combined, controlling for site
    - excluding Vietnam
    - including the data from Vietnam, but censoring all patients at the time point of the third episode in the other countries

#### 4.7.6. Incidence rate of any parasitaemia over 12 months

The analysis for the incidence rate of any symptomatic parasitaemia (*P. vivax* & any other Plasmodia) over 12 months will be calculated as described in section 4.5.1.1. The following comparisons will be performed:

- 7 day regimen versus control
- 14 day regimen versus control

The analyses will be performed in the ITT and the PP population (see 3.4.1) and as follows:

- for all sites combined, controlling for site
    - excluding Vietnam
    - including the data from Vietnam, but censoring all patients at the time point of the third episode in the other countries

#### 4.7.7. Incidence risk of any symptomatic parasitaemia over 12 months

This analysis will be performed as described in section 4.5.1.2. The first recurrent **symptomatic** episode of any parasitaemia will be treated as failure endpoint. Patients presenting with any asymptomatic parasitaemia will be censored at the day or occurrence.

Comparisons will be made between the three regimens. The analyses will be performed in the ITT and the PP population (see 3.4.1) for all sites combined, controlling for site.

#### 4.7.8. Incidence risk of any parasitaemia over 12 months

This analysis will be performed as described in section 4.5.1.2. The first recurrent **symptomatic or asymptomatic** episode of any parasitaemia will be treated as failure endpoint. No parasitaemia will lead to censoring.

Comparisons will be made between the three regimens. The analyses will be performed in the ITT and the PP population (see 3.4.1) for all sites combined, controlling for site.

## 4.8. Secondary efficacy analyses (Schizontocidal Efficacy)

### 4.8.1. Schizontocidal efficacy of chloroquine and chloroquine plus primaquine at day 28 and 42

The incidence risk is the time to the first event (recurrence of *P. vivax*) and will be assessed using survival analyses (Kaplan Meier). Therapeutic efficacy of the schizontocidal treatment in the control arm will be assessed at day 28 and 42. Patients presenting with parasitaemia other than *P. vivax* will be censored at the day or occurrence. The analyses will be performed for all sites combined, controlling for site (see also section 4.5.2). The incidence risk of *P. vivax* recurrence will be compared between the control arm (chloroquine alone) and each intervention arms.

### 4.8.2. PCR-adjusted incidence risk of *P. vivax* at day 28 and 42

The incidence risk of recurrent *P. vivax* over 28 and 42 days will be assessed by survival analysis (Kaplan Meier method) (see also 4.5.1.2), but parasites with different genotypes will be censored from the analysis. If in all of the markers used at least one allele is matching in the enrolment sample and in the sample taken at the day of recurrence, the recurrence will be considered the same infection. Otherwise, it will be considered a different infection. In the event that molecular data is missing from the day of recurrence, the patient will be censored at this day without a recorded endpoint outcome (i.e., that patient will not contribute any influence on the risk estimate). Comparison will be made between the different treatment arms. The analyses will be performed for all sites combined, controlling for site.

### 4.8.3. Proportion of patients with *P. vivax* parasitaemia on day 1, 2 and 3 after schizontocidal treatment in the control arm

Parasite clearance will be presented using the proportions of patients who remain parasitaemic on each day (days 1 to 3).

### 4.8.4. Proportion of patients with fever on day 1, 2 and 3 after schizontocidal treatment for *P. vivax* in the control arm

Fever clearance time will be presented as the proportion of patients who were febrile or had a history of fever on day 0 and who became febrile (<37.5°C) day 1, 2 and 3 with no subsequent measured fever or history of fever on subsequent daily within the next 48 hours.

### 4.8.5. Proportion of patients with *P. falciparum* parasitaemia on day 1, 2 and 3 after treatment with ACT during follow up

In the course of the follow up patients will present with P. *falciparum* infections. Those patients are treated and followed until parasite clearance. Parasite clearance of *P. falciparum* cases occurring during the follow up will be presented using the proportions of patients who remain parasitaemic on each day (days 1 to 3). Only the first episode of *P. falciparum* during follow up will be used for this analysis.

#### 4.8.6. Proportion of patients with fever on day 1, 2 and 3 after schizontocidal treatment for *P .falciparum* during follow up

As described in section 4.8.5 patients will present with P. *falciparum* infections. Fever clearance *of P. falciparum* cases occurring during the follow up will be calculated as described in section 4.8.4. Only the first episode of *P. falciparum* during follow up will be used for this analysis.

### 4.9. Additional analyses:

#### 4.9.1. Incidence risk of *P. falciparum* after *P. vivax* infection

This analysis will look into the time to the first recurrence of *P. falciparum* after the initial *P. vivax* infection and will be assessed using survival analyses (Kaplan Meier method). Patients enrolled with *P. vivax* will be included in the analyses. Patients presenting with *P. falciparum* parasitaemia after the initial *P. vivax* infection are defined as failures. Patients presenting with a recurrent pure *P. vivax* infection or any other parasitaemia other than *P. falciparum* will be censored at the time of occurrence. The analyses will be performed for all sites combined, controlling for site. Comparisons will be made between the control arm and the 7 day regimen as well as between the control arm and the 14 day regimen (see also section 4.5.2).

#### 4.9.2. Incidence risk of *P. vivax* after first *P. falciparum* infection

This analysis will look into the time to a recurrence of *P. vivax* after the first *P. falciparum i*nfection and will be assessed using survival analyses (Kaplan Meier method). Patients with *P. falciparum* infection during follow up will be included in the analyses, starting their exposure time from the time the first *P. falciparum* infection occurred. Patients presenting with *P. vivax* parasitaemia thereafter are considered failures for the purpose of this analysis. Patients presenting with a recurrent *P. falciparum* infection or any other parasitaemia other than *P. vivax* will be censored at the time of occurrence. The analyses will be performed for all sites combined, controlling for site. Comparisons will be made between the control arm and the 7 day regimen as well as between the control arm and the 14 day regimen (see also section 4.5.2).

#### 4.9.3. The distribution of *P. vivax* recurrences in the control arm

The analysis for the incidence rate of symptomatic *P. vivax* recurrence over 12 months in the control arm will be calculated as described in section 4.5.1.1 and compared between sites and regions.

#### 4.9.4. Subgroup analyses

*A priori* subgroup analyses will be performed for the incidence rate and risk of symptomatic *P. vivax* parasitaemia over 12 months (see section 4.6.1, 4.7.1, 4.7.3 ) in the following subgroups:

- By age group: to assess the treatment efficacy and risk of recurrence by age.
- By site and region: to compare the protective efficacy of primaquine in areas with different vivax relapse periodicity
- Patients with full versus partial treatment courses: to assess the impact of the duration of treatment and total amount of primaquine received.
- By schizontocidal drug: to assess whether efficacy varies with schizontocidal partner drug.

## 4.10. Safety analyses

The primary safety concerns regarding primaquine use pertain to the risk of haemolysis and gastroenterological adverse events. All safety analyses will be assessed in patients whose treatment remains blind; once treatment is un-blinded (i.e. in the case of five or more episodes of *P. vivax* malaria) then these patients will be censored from further safety analyses.

### 4.10.1. Risk of Haemolysis

#### 4.10.1.1. Incidence risk of severe anaemia or transfusion

Safety of primaquine treatment in the 7 day and the 14 day regimen will be compared by calculating the incidence risk of severe anaemia (Hb<7g/dl) and/or blood transfusion within the 12 month follow up. Incidence risk will be calculated as described in section 4.5.1.2. using the Kaplan-Meier method. Patients who develop severe anaemia will be considered failures. Patients lost to follow up or with missing measurements will be censored at the time of the last visit (see 3.3.7). Comparisons will be performed between the 7 day and 14 day regimen and the G6PDd and the control arm by comparing the relative hazards (Hazard Ratio (95% CI)) estimated from a Cox regression analysis with stratification for study site.

#### 4.10.1.2. Incidence risk of drop in Hb >5g/dl

Safety of primaquine treatment will be compared by calculating the incidence risk of an acute fall in Hb >5g/dl within 7 days of starting of primaquine/placebo treatment. Incidence risk will be calculated as described in section 4.5.1.2 using the Kaplan-Meier method. Patients with an acute drop of Hb >5g/dl will be considered as failures. Patients lost to follow up or with missing measurements will be censored at the time of the last visit (see 3.3.7). Comparisons will be performed between the 7 day and 14 day regimen and the G6PDd and the control arm.

#### 4.10.1.3. Hb level day 3 and day 7

For changes in Hb concentration between baseline and day 3 or day 7, linear mixed-effects modelling will be performed with an interaction term between treatment group and time to assess if changes in Hb vary between the intervention arms and the intervention and control arms, with random effects for the intercept and slope. Comparisons will be made between the intervention arms, the intervention arms and the control group and between the G6PDd arm and the control group.

#### 4.10.1.4. Hb nadir after treatment and the median time at which it occurs

The Hb nadir will be defined as the minimum Hb measurement during 28 days follow up after initiation of treatment. Estimated mean Hb concentrations will be compared between the intervention arms and also between each intervention arm and the control group using multivariable linear regression with adjustment for site. The median time from enrolment until the nadir is reached will be calculated for the different treatment groups and compared using multivariable linear regression with adjustment for site.

#### 4.10.1.5. Area under the curve

The area under the curve will be calculated assuming that a patient's normal Hb is reflected by their Hb on day 28[3]. Initial exploration will define whether day 28 or an alternative day is the most appropriate measure. The area under the curve will be presented for the following periods: day 0-28, day 28-365 and day 0- day 365. The estimated mean area under the curve will be compared between the intervention arms and also between each intervention arm and the control group using multivariable linear regression with adjustment for site.

### 4.10.2. Risk of Adverse Events

#### 4.10.2.1. Risk of acute vomiting

Proportion of patients with vomiting within 1 hour of administration of a dose will be calculated for each arm. The absolute difference between the intervention arms and between each intervention arm and the controls will be presented.

#### 4.10.2.2. Risk of gastroenterological events

Proportion of patients with nausea, vomiting, abdominal pain or vomiting will be calculated for each arm. Proportions for each symptom will be calculated separately. The absolute difference between the intervention arms and between each intervention arm and the controls will be presented.

#### 4.10.2.3. Overall risk of Adverse Events (AE) and Serious Adverse Events (SAE)

The proportion of patients with one or more adverse events and serious adverse events within 7, 14 and 42 days of their primary treatment will be presented. Events will be divided into related and unrelated events in regards to the treatment. The absolute difference between treatment groups in the proportion of patients (two-sided 95% CI) with AEs and SAEs will be calculated for those time points.

#### 4.10.2.4. Adherence

The proportion of patients completing a full course of observed primaquine therapy will be calculated for each arm. The absolute difference between the intervention arms and between each intervention arm and the controls will be presented.

## 4.11. Pharmacokinetic analyses

### 4.11.1. Chloroquine

The schizontocidal efficacy of chloroquine depends on adequate absorption of drug. For long half-life drugs the day 7 concentrations are a good predictor of the drug exposure (i.e. area under the drug concentration versus time profile).

Mean drug concentration of CQ will be assessed at day 7 as a measure of drug exposure. For patients that fail (defined as a recurrence of P. vivax) during the follow up the mean (95%CI) drug levels at day 7 and the day of recurrence will be presented and mean day 7 measurements

[3] Adverse effects in patients with acute falciparum malaria treated with artemisinin derivatives. Price R *et al.* Am J Trop Med Hyg. 1999 Apr;60(4):547-55. Review.

compared using the two-sample t test. The proportion of patients with recurrences that present with any CQ concentrations and those without CQ in their peripheral blood will be tabulated. Further the proportion of patients with levels ≥100ug/ml and the proportion of patients <100ug/ml will be presented. Patients with adequate drug levels at the day of recurrence are indicative to carry CQ resistant parasites.

#### 4.11.2. Primaquine

Primaquine has a short half-life, whereas its metabolite Carboxy-Primaquine stays longer in the peripheral blood stream. Samples will be taken on day 7 and 13/14. The analyses will aim to establish the normal concentration of Carboxy-Primaquine after 7 and 14 days of supervised primaquine treatment in the two intervention arms and the control arm (e.g. present a standard curve of Carboxy-Primaquine over 14 days supervised treatment).

In addition we will perform multivariable logistic regression to understand the relationship between drug concentration at day 7 and 14 and the binary outcome at 12 months, radical cure (yes versus no) with adjustment for site.
