# Supplementary material for: Improving the radical cure of vivax malaria (IMPROV): a study protocol for a multicentre randomised, placebo-controlled comparison of short and long course primaquine regimens
Source: BMC Infect Dis. 2015 Dec 7;15:558. doi: 10.1186/s12879-015-1276-2 (PMC4672497; doi:10.1186/s12879-015-1276-2)
Supplement: Additional file 2: — Statistical Analytical Plan (SAP), version number 1.0. (PDF 330 kb) [file 12879_2015_1276_MOESM2_ESM.pdf]

	**Name**	**Signature**
**Written by**	Bob Taylor	
**Approved by**	Ric Price	

Introduction

This Standard Operating Procedure (SOP) outlines the approach to managing suspected haemolysis in trial participants. The key points are repeated in a one-page flowchart for clarification. There are two annexes that contain additional information on the symptoms, signs and investigation of anaemia and drugs that can cause haemolysis or interact with primaquine.

The clinical judgement of the attending clinician and patient safety should always come first, regardless of the trial protocol. Each site varies in capacity, hence management and interventions should be adapted to local clinical practice.

Any concerns should be raised by the principal investigators at each site with the trial co-ordination team in Bangkok.

Background

Haemolysis in acute malaria is inevitable, the mean haemoglobin concentration usually falling within the first week, with recovery usually taking 28-42 days. The use of primaquine in patients with *P*. *vivax* infection can cause additional haemolysis particularly in patients with glucose-6-phosphate dehydrogenase (G6PD) deficiency; the predominant mechanism for this is intravascular haemolysis. Clinicians need to be vigilant for the signs and symptoms of haemolysis and the risk of trial subjects becoming anaemic.

Severe haemolysis and anaemia in a patient in the IMPROV study is likely to result from a variety of factors, including: the severity of G6PD enzymatic deficiency, the dose of primaquine, the degree of parasitaemia and nutritional status.

Identification of acute haemolysis

At each clinical encounter the clinician should exclude the possibility of significant haemolysis. This includes:

1. ***Detailed history and examination***: including a history of dark urine, jaundice, breathlessness and other symptoms of anaemia (see Annexe 1). A full drug history should be taken looking in particular for the use of drugs and herbal remedies that can cause haemolysis or interact with primaquine (see Annexe 2).

2. ***HemoCue haemoglobin measurement:*** this should be compared against previous haemoglobin results, particularly the baseline value prior to starting primaquine, to gauge the degree and speed of decline.

3. ***Hillmen urine colour estimation for haemoglobinuria***: urine should be placed in a clear glass container and held up against a white piece of paper, in a well illuminated area, before estimating the colour compared to the Hillmen Colour Chart. Urine colour estimation should be carried out as soon after voiding as possible. A score of 5 or above is considered evidence of haemoglobinuria.

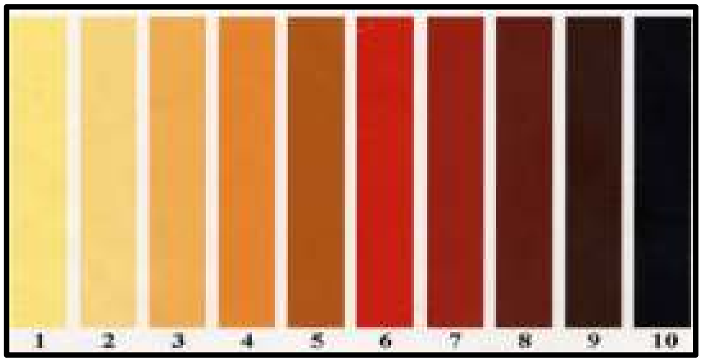

Hillmen Urine Colour Chart

4. ***Repeat G6PD fluorescent spot test:*** if on the basis of clinical assessment, haemoglobin measurement and urine colour assessment for haemoglobinuria, it is considered that haemolysis may be occurring. All patients in the G6PD normal arm of the study should have a repeat G6PD fluorescent spot test to confirm correct allocation to the G6PD normal arm.

5. ***Intensive review***: any concern should prompt increased frequency of review or admission to hospital for monitoring at the discretion of the attending clinician.

Based on this assessment, patients should be categorised into:

i) those in whom there is currently no major concern for haemolysis.
ii) those who have indicators of clinical concern but who do not constitute severe haemolysis
iii) those with severe haemolysis or anaemia

Definitions

Haemolytic Serious Adverse Event (SAE):

Any one of the following defines a haemolytic SAE:

1. *Haemoglobin of < 7g/dL OR*
2. *Anyone requiring a blood transfusion OR*
3. *Macroscopic haemoglobinuria (Hillmen ≥ 5) PLUS a fractional drop in haemoglobin ≥ 25% from baseline*

Warning signs:

Patient does not fulfil criteria for SAE but has at least one of the following:

1. *Macroscopic haemoglobinuria (Hillmen ≥ 5) OR*
2. *Fractional haemoglobin fall ≥ 25% vs. baseline at randomisation* OR
3. *Significant clinical concern with regard to anaemia or haemolysis assessed by the attending clinician.*

Acute Clinical Management

Initial management

- patients should be assessed clinically by the attending clinician, and
- subsequent primaquine/placebo doses should be withheld pending further review

Further management

All patients with a haemolytic SAE or warning signs must be have close clinical monitoring including:

- a daily clinical review
- daily haemoglobin measurement until evidence of haemoglobin recovery
- daily Hillmen urine colour estimation.
- additional more frequent review and/or admission to hospital, as indicated
- repeat G6PD testing
- the Bangkok coordination team should be informed as early as possible

At each review, a decision must be made to:

- continue to withhold primaquine/placebo or
- re-dose primaquine/placebo or
- stop primaquine/placebo completely

The decision to retreat subsequent *P. vivax* infections with primaquine in patients who have experienced an episode of acute haemolysis, is at the discretion of the attending clinician and is **not** a protocol requirement. Patients who have had an episode of severe acute haemolysis related to primaquine use, are at significant risk of further haemolysis on reexposure to primaquine or other medications causing oxidative stress. Hence the decision to redose should be made after assessing the risks and benefits and discussing these with the patient/guardian.

The following are suggested guidelines :

If the haemoglobin falls by ≥ 1 g/dL in the preceding 24 hours: primaquine/ placebo should continue to be withheld and the patient continued to be reviewed closely.

If the haemoglobin is stable or falls by < 1 g/dL in the preceding 24 hours: the patient can be re-dosed with primaquine/placebo, at the discretion of the attending clinician. Further daily monitoring should be undertaken for at least 3 days. Patients should continue as per protocol to complete 12 months of follow up.

If the patient has clinical decompensation associated with recent primaquine use and/or anaemia: then the patient should be admitted to hospital for blood transfusion. The current primaquine/placebo regimen should be stopped and the need for unblinding should be discussed with the Bangkok coordination team.

Repeat G6PD testing

Patients in the G6PD normal arm with a haemolytic SAE or warning signs should have their G6PD status rechecked immediately. Samples should be taken prior to blood transfusion (since transfusion can mask G6PD deficiency) as well as 6 weeks after the resolution of the severe acute haemolysis (or 12 weeks after the receipt of a blood transfusion).

Subsequent Clinical Management

Patients without clinical compensation who restarted their primaquine/placebo treatment without further deterioration should complete their primaquine/placebo and continue in the study as per the study schedule.

If the patient's primaquine/placebo regimen is stopped, then referral should be made to the Bangkok coordinating team to discuss unblinding. All patients should be encouraged to remain in the study and continue to be followed up, including those patients who have been unblinded.

The follow up period remains the same i.e. one year after the first dose of study drug was given. Continue to fill in the usual case record forms.

If patients develop subsequent symptomatic recurrent *P. vivax* parasitaemia, they should be treated with chloroquine or ACT and considered for primaquine regimen on a case by case basis as per local recommendations.

MANAGEMENT OF ACUTE HAEMOLYSIS

Any clinical concern of acute haemolysis (e.g. symptoms of anaemia, dark urine, clinical jaundice or falling haemoglobin) should be trigger the following:

1. A thorough history and examination
2. Immediate HemoCue haemoglobin measurement
3. Hillmen urine colour estimation for haemoglobinuria
4. Repeat G6PD test if randomised to the G6PD normal arm to confirm correct randomisation
5. Contact Bangkok coordinating team

INDICATIONS TO STOP PRIMAQUINE /PLACEBO

Either of the following:

1. Referred for blood transfusion
2. Anaemia (Hb<7g/dl) with clinical decompensation

INDICATIONS TO WITHOLD PRIMAQUINE/ PLACEBO:

Any one of the following:

1. Hb <7 g/dL & daily activities unaffected
2. Macroscopic haemoglobinuria (Hillmen ≥ 5)
3. Fractional haemoglobin fall ≥ 25% from baseline
4. Other significant clinical concern of anaemia or haemolysis

Continue to assess daily or more frequently and manage as indicated or until resolved.

Refer to Bangkok coordinating team to discuss the need to unblind

Continue routine follow up for **FULL** year from randomisation.

Daily clinical review and Hb measurement

If Hb falls ≥ 1 g/dL over preceding 24 hours:

Withhold primaquine/placebo

Continue review and repeat Hb daily until the patient can either recommence primaquine/placebo or needs referral for blood transfusion

If Hb stable or drop over preceding 24 hours is < 1 g/dL:

Re-dose with primaquine/placebo (at the discretion of the attending clinician)

Review and repeat Hb daily until Hb stable for 3 days.

SAEs NOTIFICATION WITHIN 24 HOURS FOR:

1. Hb < 7 g/dL
2. Referral for blood transfusion
3. Macroscopic haemoglobinuria (Hillmen ≥ 5) + fractional haemoglobin fall ≥ 25% from baseline

AE FORM TO BE COMPLETED

1. All patients who have primaquine withheld for any reason

Further guidelines for the management of patients with a haemolytic SAE

Patients with a haemolytic SAE and clinical decompensation should be admitted to a hospital with a blood transfusion service and managed according to local protocols.

The key principles of managing acute drug-induced haemolysis include the following:

1. Stopping all drugs known to cause haemolysis
2. Comprehensive clinical evaluation and intensive monitoring, including vital signs, fluid balance and frequent assessment for haemoglobinuria
3. Monitoring of haemoglobin and renal function
4. Blood transfusion if indicated

It is recognised that blood transfusion protocols and practices vary between different sites. The following are offered as suggested indications for transfusion:

Haemoglobin < 7 g/dL and clinical decompensation due to anaemia:

- breathless at rest
- breathlessness on exertion over a short distance
- severe fatigue/lethargy/dizziness
- prostration
- signs of cardiac failure
- cardiac chest pain

Additional Investigations

In addition to clinical samples taken for the acute management of haemolysis, the following samples should be taken if possible from patients with severe acute haemolysis for the investigation of the factors contributing to haemolysis:

- *1 x 5ml EDTA blood tubes*: for full blood count (2ml), Coomb's (direct agglutination test) (1ml), further G6PD testing, other enzymopathies (metHb reductase, glutathione reductase and pyruvate kinase deficiency).; 500ul whole blood should be stored at -20°C for genotyping, 1.5ml stored at -80°C in glycerol-based protocol quantitative assay of G6PD and other enzyme activities.
- *1 x 5ml Lithium heparin blood tube*: (1ml) Na^+, K^+, creatinine, urea, LDH, AST, ALT, total & free / unconjugated bilirubin, (1ml) haptoglobin and cell free haemoglobin concentration (-20°C), (1ml) primaquine, carboxyprimaquine and chloroquine drug concentrations (frozen at -80°C), (1ml) markers of oxidative stress. (This tube should be promptly cooled after collection, centrifuged at 1500 G for 5 mins, and plasma then frozen at –80°C).
- *1 x 10ml Urine in white topped tube (no additive) container*: ideally midstream urine, promptly freeze 2 x 1ml aliquots at –80°C.

Additional study requirements

Adverse event

An adverse event form must be filled for all patients who have their primaquine/placebo stopped for whatever reason. The following are examples of haematological AEs relevant to this SOP:

- haemoglobin of < 7g/dL
- requirement for a blood transfusion
- severe haemolysis
- clinical concern due to haemolysis
- clinical concern due to anaemia

Add in the necessary clinical details e.g. the patient had macroscopic haemoglobinuria and a fall in Hb of 25% compared to baseline (Day 0 Hb 10, Day X Hb 7.5 g/dL) and felt more tired than usual.

The patient noticed he was short of breath when walking to work and his Hb had fallen from 9.5 g/dL at baseline of Hb to 8 g/dL on Day X.

Serious adverse event

As defined above the following are SAEs:

- Haemoglobin of < 7g/dL
- Anyone requiring a blood transfusion for haemolysis
- Macroscopic haemoglobinuria (Hillmen ≥ 5) PLUS a fractional drop in haemoglobin ≥ 25% from baseline

Please follow the SAE SOP for reporting the SAE within the necessary time frame.

Questions

Any questions regarding study processes should be addressed to:

MORU Study Coordinator	Bob Taylor<br>Officer phone: +66 2 203 6373<br>E-mail: Bob@tropmedres.ac
MORU Pharmacovigilance officer	Prayoon Yuentrakul<br>Office phone: +66 2 203 6318<br>E-mail: Prayoon@tropmedres.ac

Annexe 1

Symptoms and signs of anaemia

Symptoms:

- Breathlessness
- Fatigue
- Faintness
- Palpitations
- Backache
- Headache
- Chest pain

Passing dark coloured urine (if haemolysis is the cause)

Physical signs:

- Tachycardia
- Tachypnoea
- Palmar and conjunctival pallor
- Systolic flow murmur
- Shock
- Signs of cardiac failure
- Dark coloured urine (if haemolysis is the cause)
- Jaundice (if haemolysis is the cause)

Laboratory features of haemolytic anaemia:

- Falling haemoglobin concentration/haematocrit
- Heinz bodies, bite cells and polychromasia on blood film
- Increasing reticulocyte count (causing mild macrocytosis)
- Increased LDH
- Increased bilirubin (unconjugated)
- Reduced haptoglobins
- Increased urinary urobilinogen
- Haemoglobinuria

Some of these symptoms and signs can be related to malaria, so a careful evaluation is essential.

Annexe 2

Drugs that cause haemolysis or interact with primaquine

If patients need to be treated for an intercurrent illness potentially haemolytic drugs should be avoided in patients with G6PD deficiency. These include:

- Dapsone
- Sulphonamides
- Methylene blue
- Nalidixic acid
- Nitrofurantoin
- Niridazole
- Traditional medicines
- Ciprofloxacin, norfloxacin

The following drugs should also be avoided since these may increase the exposure of primaquine, chloroquine, piperaquine and lumefantrine metabolism by inhibiting the cytochrome (CYP) 450:

- Ketoconazole, itraconazole
- Cimetidine
- Grapefruit juice
- Erythromycin
- Ritonavir
