# Supplementary material for: Improving the radical cure of vivax malaria (IMPROV): a study protocol for a multicentre randomised, placebo-controlled comparison of short and long course primaquine regimens
Source: BMC Infect Dis. 2015 Dec 7;15:558. doi: 10.1186/s12879-015-1276-2 (PMC4672497; doi:10.1186/s12879-015-1276-2)
Supplement: Additional file 3: — Ethical considerations for the IMPROV study. (PDF 241 kb) [file 12879_2015_1276_MOESM3_ESM.pdf]

Ethical Considerations

The main risk of participating in the trial is related to the potential undesirable effects of primaquine therapy. The most severe, known adverse effects of primaquine are associated with G6PD deficiency. By excluding G6PD deficient patients from randomisation and treating them with recommended weekly primaquine, the risk of acute haemolytic anaemia will be minimised. The benefit of participation is for patients randomised into the PQ arms to receive treatment against relapse.

At each routine follow up all patients will complete a clinical questionnaire and have a finger prick sample for measurement of haemoglobin concentration and blood film examination. Participants with any clinical complaints suggestive of malaria will have their blood films read immediately and those detected to have peripheral parasitaemia will be treated immediately. Asymptomatic carriage is common (~5-10%) in malaria coendemic settings, particularly in partially immune adults, and normally goes undetected and untreated. To reflect the usual clinical scenario asymptomatic parasitaemia will not be actively detected in the study participants; instead the blood film of these individuals will be stored and read in batches at a later date. The close follow up and prompt provision of healthcare of our study population will ensure that if these patients become symptomatic, they will be detected and treated promptly.

Another important ethical consideration in this trial is the proposed withholding of primaquine therapy in the control group. At some study sites (Pakistan, Ethiopia) this is standard of care – governments do not recommend primaquine therapy fearing more harm than benefit in doing so in unscreened patients. However, at all other sites primaquine therapy is recommended with expressed warnings on toxicity in G6PDd patients. G6PD testing is advised prior to commencing primaquine therapy, but in practice it is almost invariably omitted. Each governing IRB must carefully consider the withholding of primaquine and be assured of both the necessity and safety of doing so.

Primaquine has been the only drug available for the radical cure of vivax malaria since 1952 and yet its efficacy has yet to be addressed in a standardized and systematic manner [1]. The reasons for this knowledge gap are related to the technical difficulty of the clinical assessment of anti-relapse efficacy in patients remaining in malaria endemic zones. Experimental challenge trials in volunteers in non-endemic zones (e.g. North America) can't replace trials in endemic settings and come with their own ethical and other challenges [2, 3]. Secondly, there has been an inappropriate perception that vivax malaria is a relatively benign public health problem [3, 4]. Lastly the administration of primaquine in G6PD deficient individuals can potentially trigger episodes of haemolysis. Consequently current World Health Organization guidelines continue to recommend the administration of primaquine

spread out over 14 days. This is an impractical regimen that neither national malaria control programs prioritize, nor is it adhered to by physicians or the patients. It is becoming increasingly apparent that a safe and effective therapy against relapse is required to eliminate vivax malaria in a large population (between 100-400 million clinical attacks annually)[5, 6].There is a broad consensus that the efficacy of primaquine, a 60-year-old drug should be urgently and rigorously assessed in randomized controlled trials (RCTs).

The Problem: A major challenge in estimating the efficacy of primaquine comes from our inability to distinguish relapsing from new *P. vivax* infections. Secondly the timing and rate of relapse vary across regions [7]. In temperate regions (e.g. Korea) there may be as few as a single relapse in 10% of vivax patients after 8 months or more. In contrast there is a very high risk (>80%) in equatorial regions (e.g. Indonesia) beginning as early as 21 days following the primary infection [8]. Without understanding the timing and risk of local relapse, it is impossible to estimate antirelapse efficacy. An example of this is exemplified by the Indian treatment guidelines for vivax malaria which recommended until recently the administration of a 5 day course of primaquine (15mg per day) [9]. The relapse rate with such a 5 day course was found to be around 10% and was considered acceptable by international standards. It only became obvious through RCTs comparing the 5-day regimen to patients not receiving primaquine that relapse rates between the groups were indistinguishable, and thus the 5 day regimen in effect had zero efficacy [9]. A control group is equally important for the assessment of the tolerability and safety. Primaquine can cause gastrointestinal upset and in some people with G6PD deficiency it can trigger haemolysis [10]. It is not possible to estimate how much of these adverse events is attributable to the administration of primaquine and how much to the malaria itself causing red cell rupture and loss of red cell mass [5].

The Solution: A recent comprehensive review of the use of primaquine has documented all published primaquine clinical trials since 1954 [11]. The review came to the conclusion that a prerequisite for the interpretation of primaquine efficacy trials is a control arm in which patients do not receive primaquine treatment. The natural rate of recurrence and reinfection in the no-primaquine group effectively serves as the only credible denominator from which one can estimate primaquine efficacy. Such studies are referred to as randomized, relapse-controlled clinical trials (RRCTs).

The Ethical Dilemma: In many vivax endemic countries, national malaria treatment guidelines advocate the use primaquine. Hence patients in the control arm will not receive

the potential benefit of primaquine therapy. This situation requires a careful review with special consideration of the following factors:

1) Globally vivax malaria causes a considerable disease burden. Millions of people live in vivax malaria endemic regions and are at risk for malaria [12]. Treatment for recurrent vivax episodes in the form of primaquine is available, but the optimal use of the drug is unknown and treatment is underutilized resulting in the continued transmission of vivax malaria.

2) National and WHO treatment guidelines focus on the need for prompt and effective treatment of erythrocytic stage malaria parasites but diverge markedly on radical treatment. The 2010 WHO Malaria Treatment Guidelines [13] recognise that current recommendations for primaquine are based on insufficient data. These guidelines (p.51) state that in areas of sustained transmission the use of primaquine is not considered to outweigh the risks.

3) The safe administration of primaquine is generally considered to require the exclusion of G6PD deficient patients. Few health care providers in vivax endemic settings have routine access to such facilities to diagnose G6PD deficiency. In the absence of G6PD deficiency screening most health care providers are reluctant to prescribe primaquine.

4) Even when recommended, primaquine is not routinely prescribed. Reasons for this omission include safety concerns, uncertainty over efficacy and a widely held belief that relapses are unavoidable, relatively benign, and can be treated simply by repeated blood schizontocidal treatments.

5) When primaquine is prescribed it is unsupervised, poorly adhered to, and so lacks effectiveness. In fact most vivax patients globally do not receive effective antirelapse treatment.

6) In October 2011 representatives from 10 national malaria control programs in the Asia Pacific Region, including key figures on national ethics review panels attended a meeting on primaquine in Jiangsu, China [14]. There was agreement that the large majority of vivax patients never receive primaquine, that more evidence from clinical trials is needed and that a control group is critical for scientific integrity of such trials.

7) Patient safety cannot be compromised. All patients in the IMPROV studies will be treated immediately with highly effective blood schizontocidal therapy for their primary presentation and each subsequent episode. This therapy assures the cure of the acute disease. Inactive sleeper stages of *P. vivax* in liver are completely silent and are not associated with any ongoing pathology or symptoms.

8) Subjects in the IMPROV study will be actively and closely monitored for early indications of recurrent of parasitaemia and onset of illness initially at weekly and then at monthly intervals. This will ensure immediate and effective therapy against blood stage infections.

9) Use of control arms in primaquine trials is well established. The recent review of primaquine trials found 18 trials which included a control arm in which patients did not receive anti-relapse treatment [11]. Collaborators on the proposed trial are currently conducting, or have recently conducted, anti-relapse studies with control arms in Indonesia, Thailand, Afghanistan and Pakistan which were approved by local, Oxford and London School of Hygiene and Tropical Medicine ethics review boards.

The principle of equipoise demands there be reasonable doubt concerning the inferiority of a treatment arm in a clinical trial. Authoritative agencies have given guidance on such use of "inferior" therapies in clinical trials as follows:

ICH Guidance: The International Conference on Harmonization of Technical Requirements for Registration of Pharmaceuticals for Human Use (ICH) provides guidance on placebo- or no treatment-controlled trials. ICH-E10 states the following: "*… it is generally considered ethical to ask patients to participate in a placebo-controlled trial, even if they may experience discomfort as a result, provided the setting is non-coercive and patients are fully informed about available therapies and the consequences of delaying treatment… Whether a particular placebo-controlled trial of a new agent will be acceptable to subjects and investigators when there is known effective therapy is a matter of investigator, patient, and IRB judgment*."

WMA Guidance: The World Medical Association (WMA) offered the following guidance on placebo-controlled therapeutic trials*: "Circumstances where a placebo-controlled trial might be ethically acceptable even if proven therapy was available. These are:*

1) *where for compelling and scientifically sound methodological reasons its use was necessary to determine the efficacy or safety of a prophylactic, diagnostic or therapeutic method; or*

2) *where a prophylactic, diagnostic or therapeutic method was being investigated for a minor condition and the patients who received placebo would not be subject to any additional risk of serious or irreversible harm"*

CIOMS Guidance: The Council for International Organizations of Medical Sciences (CIOMS) considered placebo controls in therapeutic trials acceptable when: *1) no effective treatment exists; 2) use of placebo control entails minor risks to subjects; or 3) active control would not yield reliable results.*

Conclusion: Randomized, controlled clinical trials come with a compelling scientific methodological rational, along with clinical and public health implications. Subjects randomized to the control arms (no- primaquine) will have their acute disease promptly and effectively treated, and are therefore at no risk of serious or threatening illness. The use of a control group, whether placebo-controlled or not, should be considered fundamentally ethical provided the trial ensures that participants are fully informed before consenting to participate and the trial provides virtually complete freedom from risk of serious illness or permanent disability resulting from participation.

References:

1. Vale N, Moreira R, Gomes P: **Primaquine revisited six decades after its discovery.** *Eur J Med Chem* 2009, **44:**937-953.
2. Anstey NM, Douglas NM, Poespoprodjo JR, Price RN: **Plasmodium vivax: clinical spectrum, risk factors and pathogenesis.** *Adv Parasitol* 2012, **80:**151-201.
3. Price RN, Douglas NM, Anstey NM: **New developments in Plasmodium vivax malaria: severe disease and the rise of chloroquine resistance.** *Curr Opin Infect Dis* 2009, **22:**430-435.
4. Price RN, Tjitra E, Guerra CA, Yeung S, White NJ, Anstey NM: **Vivax malaria: neglected and not benign.** *Am J Trop Med Hyg* 2007, **77:**79-87.
5. Baird KJ, Maguire JD, Price RN: **Diagnosis and treatment of Plasmodium vivax malaria.** *Adv Parasitol* 2012, **80:**203-270.
6. Battle KE, Gething PW, Elyazar IR, Moyes CL, Sinka ME, Howes RE, Guerra CA, Price RN, Baird KJ, Hay SI: **The global public health significance of Plasmodium vivax.** *Adv Parasitol* 2012, **80:**1-111.
7. White NJ, Imwong M: **Relapse.** *Adv Parasitol* 2012, **80:**113-150.
8. White NJ: **Primaquine to prevent transmission of falciparum malaria.** *Lancet Infect Dis* 2013, **13:**175-181.
9. Galappaththy GN, Omari AA, Tharyan P: **Primaquine for preventing relapses in people with Plasmodium vivax malaria.** *Cochrane Database Syst Rev* 2007**:**CD004389.
10. Howes RE, Piel FB, Patil AP, Nyangiri OA, Gething PW, Dewi M, Hogg MM, Battle KE, Padilla CD, Baird JK, Hay SI: **G6PD deficiency prevalence and estimates of affected populations in malaria endemic countries: a geostatistical model-based map.** *PLoS Med* 2012, **9:**e1001339.

11. John GK, Douglas NM, von Seidlein L, Nosten F, Baird JK, White NJ, Price RN: **Primaquine radical cure of Plasmodium vivax: a critical review of the literature.** *Malar J* 2012, **11:**280.
12. Gething PW, Elyazar IR, Moyes CL, Smith DL, Battle KE, Guerra CA, Patil AP, Tatem AJ, Howes RE, Myers MF, et al: **A long neglected world malaria map: Plasmodium vivax endemicity in 2010.** *PLoS Negl Trop Dis* 2012, **6:**e1814.
13. http://www.who.int/malaria/publications/atoz/9789241547925/en/index.html.
14. http://apmen.org/storage/newsmedia/APMEN_Newsletter_Issue_Five.pdf.
